# Identification of favorable alleles from exotic Upland cotton lines for fiber quality improvement using multiple association models

**DOI:** 10.3389/fpls.2025.1553514

**Published:** 2025-04-16

**Authors:** Hrithik Mangla, Min Liu, Deepak Vitrakoti, Rama Vamsi Somala, Tariq Shehzad, Rahul Chandnani, Sayan Das, Jason G. Wallace, John L. Snider, Don C. Jones, Peng W. Chee, Andrew H. Paterson

**Affiliations:** ^1^ Plant Genome Mapping Laboratory, University of Georgia, Athens, GA, United States; ^2^ Department of Crop & Soil Sciences, University of Georgia, Athens, GA, United States; ^3^ Agricultural Research, Cotton Incorporated, Cary, NC, United States

**Keywords:** *Gossypium hirsutum*, exotic lines, GBS, QTL mapping, joint linkage association mapping, fiber quality, Upland cotton

## Abstract

Upland cotton (*Gossypium hirsutum*) faces the challenge of limited genetic diversity in the elite or improved gene pool. To address this issue, we explored alleles contributed by five ‘converted’ exotic lines sampling most of the undomesticated botanical races of *G. hirsutum*, in BC_1_F_2_ and F_3_ populations. Joint analysis of all populations along with population-specific analyses identified 38 unique QTL for six different fiber quality traits. At 15 of these loci, DES56 or the elite allele improved upon all the exotics. For another 15, only a single of the five exotics improved upon the elite allele, suggesting the rare alleles that may not have been sampled in the cotton domestication or improvement. At the remaining 8 QTL, multiple exotic lines contributed the superior allele, suggesting that DES56 (and by extension the elite gene pool) has chronically poor alleles at these loci. Converted strains T1046, T326, and T063 showed the highest potential for contributions to cotton fiber quality breeding programs. Upper Half Mean Length and Fiber Strength showed multiple QTL regions affecting both traits simultaneously, while the Uniformity Index showed the smallest heritability values. The estimation of pairwise genetic distances for six parental lines indicates that DES56 has a higher genetic similarity with each exotic line than the exotic lines have with each other. Most of the detected QTL were ‘minor’ (explaining less than 10% of variance) supporting the implementation of genomic selection techniques to utilize the cumulative effects of most of these QTL distributed genome-wide. Finally, some regions were consistently unfavorable for exotic introgression such as on chromosomes A13 and D09, indicating the possible genome-wide haplotypes that may combine the benefits of a history of scientific breeding of the elite gene pool.

## 1 Introduction

Cotton (*Gossypium* spp.) is one of the most important cash crops and a leading source of textile fiber. In the United States, Upland cotton production was 12.8 million bales from the harvested area of 8 million acres in 2023 (USDA- National Agricultural Statistics Service, 2023). The total export value for cotton in 2023 was $5.95 Billion in the United States.

The genus *Gossypium*, belonging to the Malvaceae family, is considered to have more than 50 species, 45 diploid (2n = 26) and seven tetraploid (2n = 52), with a basic chromosome number of 13 (Fryxell et al., 1992). The allotetraploid cotton species are believed to have been formed by hybridization of genotypes resembling modern *G. herbaceum* (A genome) and *G. raimondii* or *G. gossyipoides* (D genome) about 1-2 million years ago (Guo et al., 2014; Wendel, 1989). Only 4 *Gossypium* species are cultivated, 2 of which are allotetraploid – ‘Upland’ cotton (*G. hirsutum*) and ‘Pima’ cotton (*G. barbadense*). Upland cotton dominates commercial production owing to its higher yield, early maturity, and resistance to diseases and pests followed by Pima cotton (*Gossypium barbadense*) for its superior fiber quality.

The Upland cotton gene pool has experienced multiple genetic bottlenecks imposed by polyploidization and domestication history followed by intense selection pressure for high-yielding and early maturing varieties. Further, repeated intercrossing of a limited number of selected varieties eventually left breeders with limited opportunities for continual improvement of fiber quality and other traits (Hinze et al., 2016; May et al., 1995; Paterson et al., 2004). Allelic diversity ‘left behind’ in the undomesticated exotic gene pools, especially from tropical regions like Mexico and Guatemala, can offer a rich source for superior and novel alleles for improving fiber quality and other traits (McCarty et al., 1996, 1998). Primitive accessions and landraces from these regions have been converted to day-neutral type via repeated backcrosses to facilitate their use in various breeding operations (McCarty and Jenkins, 2002, 2005; McCarty et al., 1979). Previous studies have shown the benefits of the use of converted exotic race stocks in fiber quality improvement despite that agronomic traits were average or inferior to elite commercial lines (Campbell et al., 2014; McCarty et al., 1996, 2004, 2007). In such scenarios, the implementation of DNA markers is necessary to mitigate the undesirable effects of linkage drag (Campbell et al., 2014; Liu et al., 2000).

Fiber quality is a set of traits with many components, controlled by networks of genes in various molecular pathways exemplifying the quantitative nature of the inheritance of these traits (Paterson et al., 2012). In the current study, we explore 6 fiber quality traits: Micronaire (MIC), Upper Half Mean Length (UHM), Fiber Elongation (ELO), Fiber Strength (STR), Uniformity Index (UI) and Short Fiber Content (SFC). MIC is an airflow measurement that estimates fiber fineness and maturity, with lower MIC values indicating finer fiber usually preferred by the textile industry, though sometimes it could be the result of immaturity (Draye et al., 2005). UHM is the mean length of the longer half of the fibers in a sample measured in hundredths of an inch. Longer UHM lengths are preferred. STR is reported as ‘grams per tex’, indicating the force in grams required to break a bundle of fibers one ‘tex’ unit in size (the weight in grams of 1000 meters of fiber), and stronger fibers are preferable. Fiber elongation (ELO) is the percentage increase in fiber length before breaking when subjected to a specific amount of tensile force. The stretchiness of fibers is preferable, reducing breakage during processing. Uniformity Index (UI) is a ratio between the mean length and the upper half mean length of the fibers, expressed as a percentage and indicating the uniformity of fiber length in a sample. Short fiber content (SFC) is the proportion of fibers with a length less than 0.5 inches, generally inversely related to UHM, and with a minimum value preferable.

The development of high-quality reference genomes, high-density linkage maps, and high throughput phenotyping techniques amplified the acquisition of QTL information related to several fiber quality traits, serving as the foundation for marker-assisted selection and genomic selection. For example, an important QTL region validated on chromosome 25 for fiber length or upper half mean length has been introgressed from *G. barbadense* into *G hirsutum* lines (Brown et al., 2020). However, QTL data often is incongruent among different studies due to factors like environmental variation, genetic background effects, and the type and size of mapping populations used. To generate consensus QTL information from large numbers of studies, meta-QTL analyses have been conducted (Rong et al., 2007; Xu et al., 2020) and suggest a non-uniform distribution across the genome for fiber quality traits. Some of these converted exotic race stocks have also been utilized in biparental populations with SSR (Simple Sequence Repeats) markers genotyping to identify favorable alleles that could be introgressed into elite cultivars (Adhikari et al., 2017). However, the advent of high-density genotyping techniques like Genotyping-by-sequencing (GBS) (Elshire et al., 2011) and the cheap prices for sequencing present the opportunity to further accelerate the process of dissecting these QTL regions with more precision.

QTL mapping studies for fiber quality traits using advanced backcross families (Chee P. et al., 2005; Chee P. W. et al., 2005; Chen et al., 2020) helps in the simultaneous discovery and introgression of traits in the recurrent background (Tanksley and Nelson, 1996). Both linkage mapping using bi-parental populations (Chandnani et al., 2018; Chee P. et al., 2005; Kumar et al., 2019) and association mapping techniques (Ademe et al., 2017; Liu et al., 2020) have shown potential in mapping the genomic regions associated with fiber quality traits. To further increase the power and precision of mapping these QTL, joint linkage association techniques (leveraging the strength of an increased number of recombination events and sample size) could be implemented in multiple biparental populations (Myles et al., 2009) as reported using nested association mapping populations in other crops (McMullen et al., 2009; Olatoye et al., 2020).

Hence, to leverage the strengths of these high-density genotyping techniques and powerful joint linkage association mapping techniques, we describe QTL mapping in five BC_1_F_2_ and one F_3_ intermated population(s) generated using five exotic day-neutral converted Upland cotton lines sampling most of the botanical races of *G. hirsutum* (**Supplementary Table S1A**). The choice of a common elite parent was strategic: DES56 is suggested to be in the pedigrees of more elite cotton cultivars than any other line and is arguably the best single representative of the elite cotton gene pool (Suszkiw, 2010). The major objective is to identify the genomic regions in these exotic lines that can contribute the favorable alleles for fiber quality improvement in Upland cotton.

## 2 Materials and methods

### 2.1 Plant materials and phenotyping

Five BC_1_F_2_ populations, each comprised of 4 families from different BC_1_F_1_ plants; and an F_3_ intermated population of 4 F_2_-derived families resulting from intermating among different F_1_ plants, were developed using five converted exotic cotton lines namely T326, T281, T257, T063 & T1046, with elite line DES56 as the recurrent parent in all backcross populations (**Supplementary Figure S1**). The exotic lines were selected based on their estimated genetic effects for various fiber quality traits reported by previous studies (McCarty et al., 1996, 1998, 2007).

In 2012, 200 seeds per population were planted at the University of Georgia’s Plant Science Farm, including 50 seeds from each of four different BC_1_F_1_ or F_2_ (in the case of F_3_ population) plants, at 12” intervals. The final number of lines achieved for the 6 populations were 168 (T326 x DES 56), 179 (T0257 x DES 56), 180 (T1046 x DES 56), 176 (T1046 x DES 56 (IM)), 162 (T0281 x DES 56), 179 (T063 x DES 56). Hereafter, these populations will be denoted as populations 1, 2, 3, 4, 5, and 6 respectively for convenience.

BC_1_F_2:3_ progeny rows were planted in 2013, and plants from each population were selfed and harvested in bulk as the full plots or progeny rows. During the next year (2014), BC_1_F_2:4_ progeny rows were again planted at the University of Georgia’s Plant Science Farm. In all three years, the harvested lint was sent to Cotton Incorporated (Cary, NC) to measure the fiber quality parameters (MIC, UHM, ELO, UI, STR, and SFC) by the High-Volume Instrument (HVI’) system.

### 2.2 Phenotypic data analysis

All phenotypic data analyses were carried out using the R statistical tool (R core Team, 2024). Firstly, all populations were confirmed for genotypic, family, and environmental effects using the analysis of variance method.

For Broad Sense heritability estimation, a mixed linear model was implemented with genotype and environment included as the random effects (Equation 1). Variance components were extracted for all the above components along with the residual variance which were used to calculate H^2^ or plot level heritability values as follows


$$\;H^{2}=\sigma_{G}/(\sigma_{G}+\;\sigma_{R})$$

where *σ_G_* represents the variance explained by genotypic or genetic factors and *σ_R_* by residual factors or unexplained variance.

Further, the Best Linear Unbiased Predictors (BLUPs) were generated for each sample using 3 years of data for each trait using the “lmer” function in the “lme4” package-


(1) $$y=u+a_{G}u_{G}+a_{E}u_{E}+e$$

*y* represents the phenotype of each individual with *a_G_* and *u_G_* ( $u_{G}∼\;N(0,\sigma_{u_{G}}^{2})$ ) representing the individual’s genotypic factor and its corresponding random effect respectively (Equation 1). Similarly, *a_E_* and *u_E_* ( $u_{E}∼\;N(0,\sigma_{u_{E}}^{2})$ ) represent the environmental factor and its corresponding random effect, respectively (Equation 1). The symbol *u* denotes the fixed intercept and *e* is the normally distributed residual or unexplained variation (Equation 2). The final breeding values for each individual were calculated as the sum of the fixed intercept (*u*) and the BLUP values for the genotypic effect of that individual (*u_G_*) which were used for the actual association analyses. For family and population-specific analyses these models were fitted for each individual population while for the joint analysis, the above model was fitted jointly for all populations.

The predicted breeding values for all joint populations were used for correlation analyses among the traits to extract the Pearson’s correlation values mostly due to the genotypic effects, minimizing random environmental effects. Statistical parameters including mean, standard deviation and coefficient of variation were also calculated for the predicted breeding values and for actual phenotypic values for each population.

### 2.3 Genotyping and data analyses

Genomic DNA was extracted from freeze-dried leaves via a modified CTAB (cetyl trimethyl ammonium bromide) protocol (Paterson et al., 1993) and stored at -80°C. Genotyping by sequencing (GBS) libraries were constructed for 96 samples/library (Elshire et al., 2011). In brief, DNA samples were digested with *TfiI* enzyme (High-Fidelity; New England Biolabs Inc., Ipswich, MA, USA), then ligated to a unique barcode adapter. The pooled library containing 96 samples was subjected to polymerase chain reaction using 2X GoTaq Colorless Master Mix (Promega, Madison, WI, USA), and the amplified product was run on 2% agarose gel after electrophoresis where the 200-500 bp fragments were extracted and purified using a Qiagen Gel Extraction Kit (Qiagen, Hilden, Germany). The prepared GBS libraries were subjected to paired-end sequencing of 150 bp read length using BGI’s DNA Nanoball sequencing technology (DNBSEQ400-PE150).

The sequenced fastq files were confirmed for a minimum Phred score of 30, then demultiplexed for each sample using the “process-radtags” command in Stacks (Catchen et al., 2013) software, followed by the trimming of adapter sequences. The trimmed fastq files were aligned to the cotton reference genome “v3.1” (Sreedasyam et al., 2024) using the Burrows-Wheeler Alignment (BWA) tool.

GATK’s “HaplotypeCaller” plugin was used to call the variants on the sorted and indexed BAM files from the previous step and the raw VCF file was filtered for only biallelic SNPs. After performing the thinning operations, the resulting VCF file was used in “TASSELv5.2” (Bradbury et al., 2007) software to remove sites having greater than 30% missing data and progenies with >10% missing sites. Further, the sites were filtered for a minor allele frequency (MAF) of 0.10 (If a single family is segregating 1:2:1, the MAF across all populations comes out to 0.12, hence the 0.10 cutoff). The final VCF file was imputed to fill the remaining missing sites using “BEAGLE haplotype phasing” (Browning et al., 2021). Final populations deviated from the expected segregation ratios significantly, obstructing the construction of robust genetic maps. To overcome this limitation, high-density physical maps were utilized with manual elimination of any smaller sites showing inconsistency with adjacent sites. The total number of SNP markers retained after these filtering operations ranged between 2500 and 5100 distributed across the genome for each population and about 43000 for the joint population.

For association analyses of each population, three models were implemented – nested joint linkage association (nested JLM) (markers nested within family factor to identify the QTL significant within the families), non-nested joint linkage association (identifies the QTL significant across the families) (non-nested JLM), and multi-locus mixed model (MLMM) using a kinship matrix. The first two models were implemented in TASSELv5.2 (Bradbury et al., 2007) software using the stepwise plugin with an entry p-value of 0.01 and exit p-value of 0.02 along with 1000 permutations (Churchill and Doerge, 1994) to determine statistical significance thresholds appropriate for multiple comparisons. The marker R^2^ or percentage phenotypic variation (PVE) explained was calculated as the (sum of squares (actual marker association)/Total sum of squares) *100. The additive effects for each marker were calculated as the difference between the means of homozygous classes divided by two. In addition to that, the family factor was included in both models to remove any spurious associations arising due to general background effects rather than specific marker-trait associations.

For the third model, the “GAPIT” (Lipka et al., 2012) package in R (R core Team, 2024) was implemented where forward and backward regression was used as in the above two models. For controlling the population structure, instead of including the family factor, the kinship matrix was utilized here. The final p-value threshold was set to 0.00001 in this case which approximates the value (Haynes, 2013) obtained via Bonferroni correction (P-value < 0.05) for multi-testing in each population.

For joint analysis of all 6 populations, the non-nested JLM model was implemented in TASSEL v5.2 by using the stepwise plugin as follows-


(2) $$y=X_{G}B_{G}+a_{p}u_{p}+a_{f}u_{f}+e$$

where *X_G_* denotes the design matrix for SNP or marker effects, *B_G_* denotes the corresponding fixed marker effects, *a_p_* and *a_f_* signify the population and family of each individual respectively with the corresponding effects of *u_p_* and *u_f_*, and e denotes the residual or unexplained variation (Equation 2). In the case of population-specific analyses, *a_p_* and *u_p_* were excluded from the model.

QTL intervals were defined by implementing the original models without stepwise regression and extracting the physical positions upstream and downstream of the original associations where p-value <0.001 in the case of population-specific analyses and p-value < 0.0001 in the case of joint analysis. Intervals add extra confidence to significant QTL as compared to single marker associations which could be artifacts sometimes.

## 3 Results

### 3.1 Genetic diversity among the parental lines

**Figure 1:**
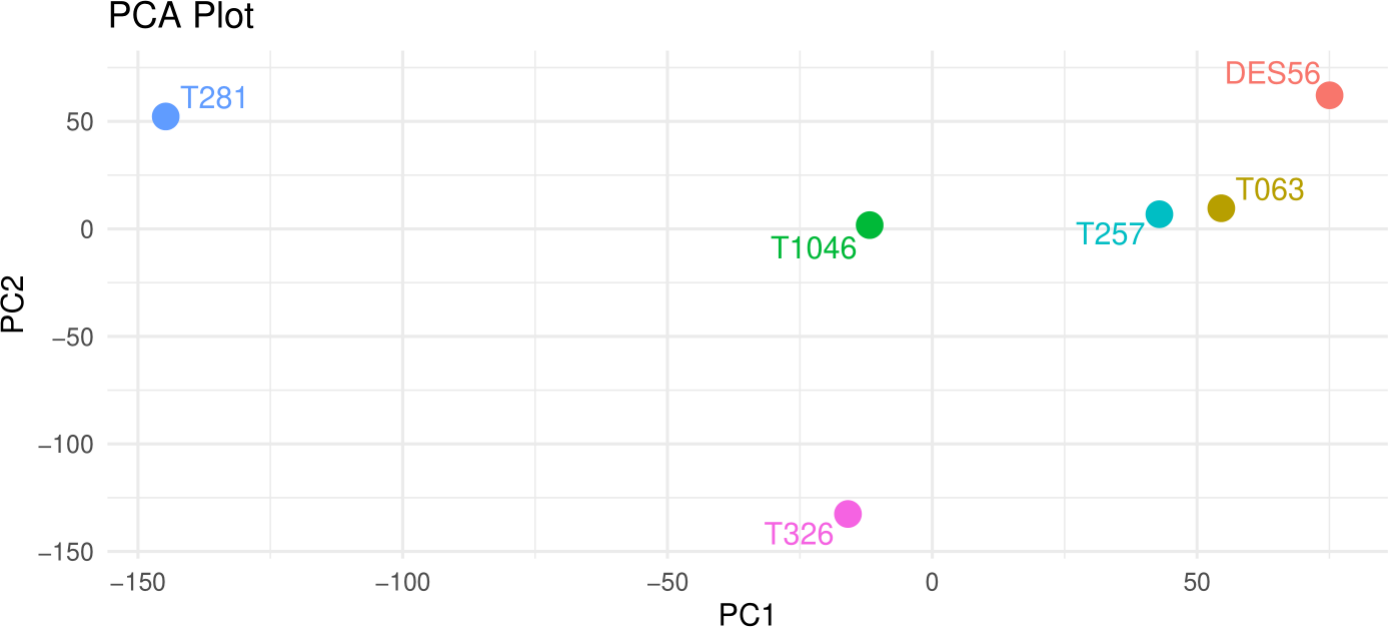
Principal Component Analysis of the 5 exotic lines and DES56 based on 140564 SNP marker sites distributed across the genome, PC- Principal Components, PC1 (x-axis) explained about 29% of the variation while PC2 (y-axis) explained about 22%. DES56 denotes the common parent while the T prefix indicates the converted forms of the exotic lines used in this study.

Principal component analysis (PCA) plots were based on 140564 SNP markers representing the variants detected among genomic sequences of the 6 parental lines, for which at least one of the six parental lines had a different allele (**Figure 1**). The PCA plot indicated elite ‘DES56’ to be clearly differentiated from all exotic lines (**Figure 1**). T257 and T063 were relatively close to each other and DES56, while T281 and T326 were comparatively far from DES56. The pairwise genetic distances (1-p(IBS)) (p(IBS) = the probability that alleles drawn at random from two individuals at the same locus are the same) among DES56 and the other 5 exotic parents calculated using these 140564 SNPs (**Table 1**) are consistent with PCA analysis suggesting two lines (T257 and T063) to be relatively closer than the remaining 3. Interestingly, for almost all the exotic lines, the relative genetic distance between DES56 and the exotic line was smaller than the pairwise distance between any two exotic lines (**Table 1**).

**Table 1:** Genetic distance among 6 Parental lines used in this study calculated based on 140564 SNPs or variant sites among the 6 parents and using 1-p(IBS) formula, where p(IBS) stands for the probability that alleles drawn at random from two individuals at the same locus are the same.

Taxa	DES56	T063	T1046	T257	T281	T326
DES56	0	0.286443	0.405886	0.366139	0.466471	0.414505
T063	0.286443	0	0.417102	0.384298	0.477878	0.395773
T1046	0.405886	0.417102	0	0.458553	0.481773	0.457521
T257	0.366139	0.384298	0.458553	0	0.504735	0.444363
T281	0.466471	0.477878	0.481773	0.504735	0	0.481973
T326	0.414505	0.395773	0.457521	0.444363	0.481973	0

### 3.2 Phenotypic data analyses

The parental phenotypic values for 6 fiber quality traits are provided in **Supplementary Table S1B**. Significant differences were observed among the 6 parental lines including DES56 and 5 exotic lines for all traits (p-value<0.01) (**Supplementary Table S1C**), however for the pairwise comparison Tukey’s HSD test (not shown) didn’t show a significant difference among DES56 and the other lines for most traits. To further confirm the genotypic and environmental effects, ANOVA analysis was carried out for each population (**Supplementary Table S2**). The ANOVA results showed both environmental and genotypic effects to be highly significant (p-value < 0.01) for most traits in all populations, except for UI in populations 1 and 6 (**Supplementary Table S2**).

**Figure 2:**
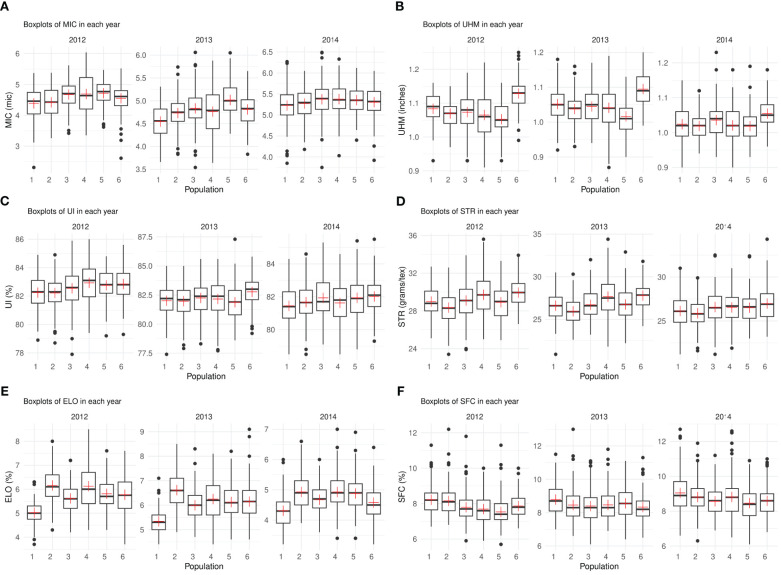
**(A-F)** – Boxplots depicting the median and interquartile range for each trait in three different years, the “+” sign indicates the mean value for each population (x-axis). Each subplot represents the phenotypic values (y-axis) for one of the six traits – **(A)** MIC **(B)** UHM **(C)** UI **(D)** STR **(E)** ELO **(F)** SFC.

For MIC, population 1 consistently showed lower mean values in all years overall (**Figure 2A**). Population 6 performed the best for UHM in all environments while population 5 had the lowest mean value for this trait in almost all years (**Figure 2B**). Populations 4 and 6 possessed maximum mean values for UI and STR in different years (**Figures 2C, D**). Population 1 had exceptionally low mean values for ELO in all years compared to other populations while 2 and 4 had the maximum mean values overall in all years (**Figure 2E**). Populations 3, 4, 5 and 6 consistently showed lower values for SFC in all environments (**Figure 2F**).

Most of the traits included in this study showed a continuous distribution approximating normality for each individual population and the joint population. The mean phenotypic breeding values for each population had a smaller range for most traits (**Table 2A**). The coefficient of variation was the highest for ELO followed by MIC and STR while UI showed the lowest coefficient of variation values within the range (**Table 2A**). The predicted breeding values for the joint population also showed similar trends among all traits (**Table 2A**). Statistical parameters for the actual phenotypic values closely resembled those for the predicted breeding values. However, a large difference between actual phenotypes and the predicted breeding values for standard deviations and coefficients of variation values indicated a higher proportion of environmental and residual variances (**Table 2B**).

**Table 2:** (A, B) Summary of the trait statistical parameters calculated for each population to depict the range, values in the bracket represent the statistical parameters calculated for the joint population, and the upper table (2A) summarizes the parameters for calculated breeding values excluding environmental effect while the lower table (2B) shows the parameters for actual trait values.

2A)			
Trait	Mean Range	Standard Deviation Range	Coefficient of Variation Range
MIC	4.725-5.029 (4.926)	0.077-0.269 (0.189)	0.016-0.055 (0.039)
UHM	0.994-1.092 (1.043)	0.014-0.039 (0.038)	0.014-0.038 (0.036)
UI	81.435-82.541 (82.071)	0.053-0.665 (0.384)	0.001-0.009 (0.005)
STR	25.447-28.232 (27.164)	0.471-1.231 (1.145)	0.017-0.049 (0.043)
ELO	4.913-6.156 (5.56)	0.164-0.49 (0.444)	0.034-0.086 (0.08)
SFC	8.195-8.729 (8.424)	0.124-0.381 (0.284)	0.015-0.046 (0.034)
2B)			
Trait	Mean Range	Standard Deviation Range	Coefficient of Variation Range
MIC	4.718-5.03(4.901)	0.448-0.589(0.532)	0.09-0.119(0.109)
UHM	1.029-1.093(1.046)	0.045-0.064(0.058)	0.043-0.061(0.056)
UI	81.886-82.541(82.102)	1.204-1.5(1.333)	0.015-0.019(0.017)
STR	26.713-28.214(27.278)	1.935-2.437(2.25)	0.073-0.088(0.083)
ELO	4.9-5.897(5.593)	0.653-0.992(0.924)	0.134-0.174(0.166)
SFC	8.189-8.703(8.402)	0.806-1.06(0.994)	0.098-0.128(0.119)

**Figure 3:**
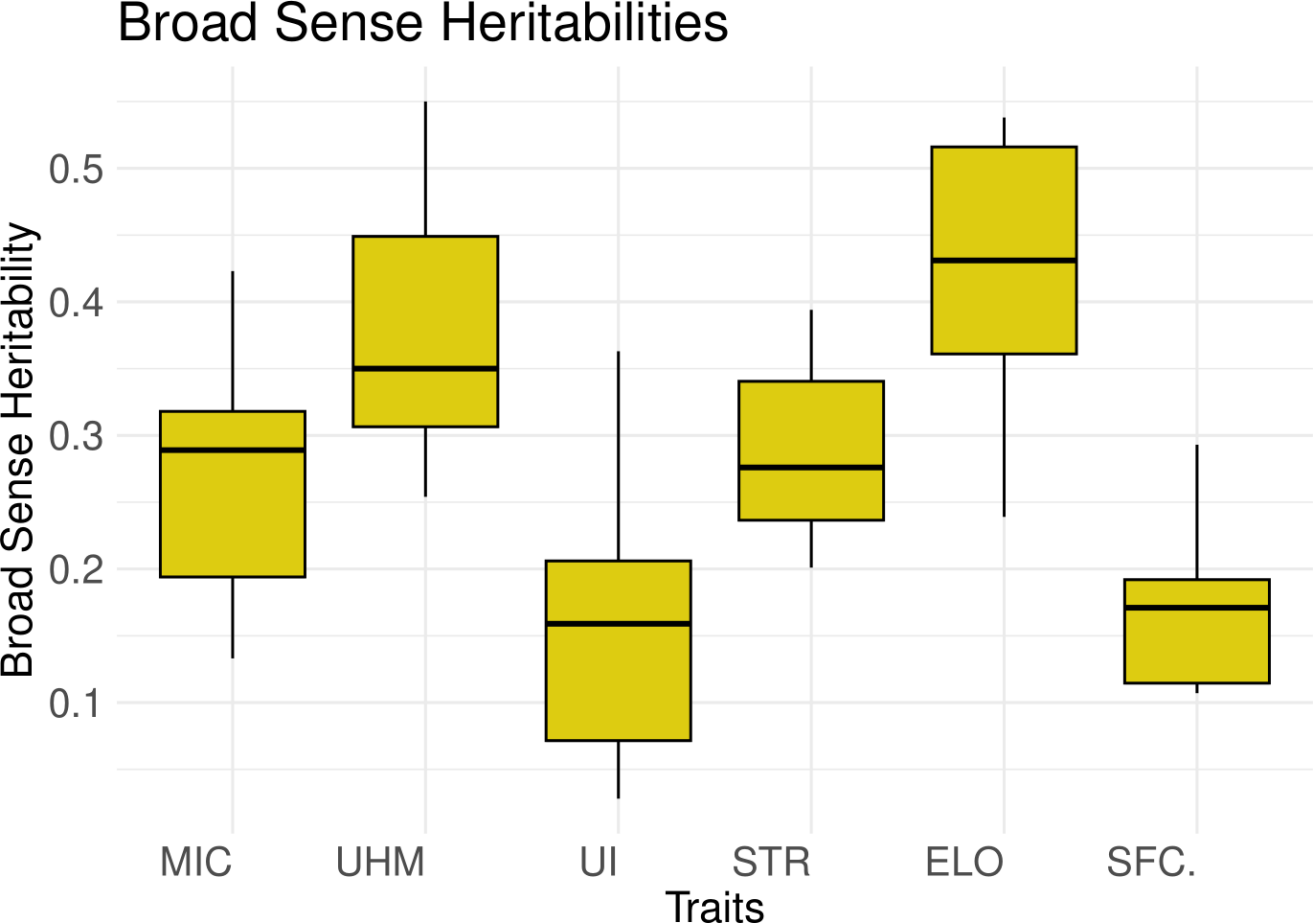
Box plots for the broad sense heritability values (y-axis) calculated for all traits (x-axis) in 6 populations depicting the median and interquartile range.

The median broad sense heritability (H^2^) or plot level heritability values are low to moderate for all traits across all 6 populations (**Figure 3**, **Supplementary Table S6**). UHM and ELO showed the highest values while UI had the lowest median H^2^ values, consistent with the ANOVA and coefficient of variation results.

**Figure 4:**
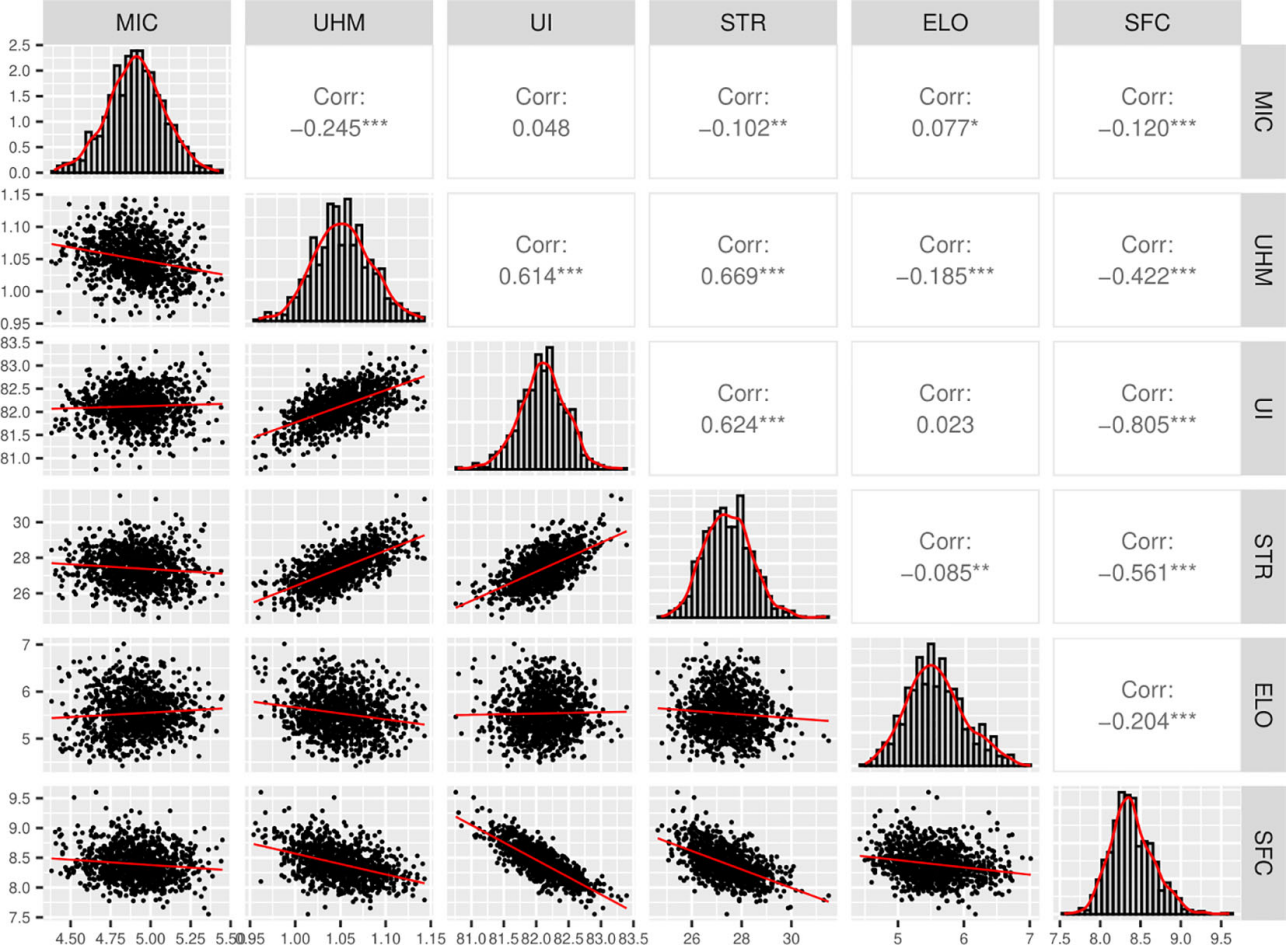
Correlation and density plots for 6 fiber quality traits, Pearson’s correlation values were calculated from breeding values predicted for all individuals jointly across the 6 populations, “*”, “**” and “***” denotes significance at p-value < 0.05, 0.01 and 0.001 respectively, “Corr” indicates the Pearson’s correlation coefficient value.

Pearson’s correlation values between the traits are based on the breeding values predicted jointly for all the individuals across 6 populations (**Figure 4**). There was a significant positive correlation (p-value < 0.001) between MIC and ELO (**Figure 4**). UHM had a significant positive correlation with STR and UI but a significant negative correlation with SFC, ELO and MIC (**Figure 4**). Similarly, UI and STR had a significant positive correlation with each other but a negative correlation with SFC (**Figure 4**). Overall, UHM, STR, and UI showed a significant positive correlation with each other but significant negative correlations with the other 3 traits in most cases (**Figure 4**).

We also calculated the number of families out of 24 (4 families in each of the 6 populations) showing superior transgressive mean breeding values for all the traits compared to the parental values (**Supplementary Table S7**). Overall, population 1 had the highest number of families showing positive mean transgression, followed by population 5 while population 6 had no family showing positive mean transgressive values (**Supplementary Table S7**). Among the six traits, UI and SFC showed the greatest number of families showing superior mean transgressive values among all populations while ELO showed the least (**Supplementary Table S7**).

### 3.3 Within families and across families or population-specific QTL analysis

QTL were designated as q{trait name}_{population name}_{chromosome}_{QTL number on that chromosome (in case of two or more QTL on the same chromosome for the same trait)}. A total of 52 QTL were identified collectively using 3 methods for all 6 populations, out of which 34 were ‘unique’ (inferred to be the same QTL detected by two or more methods) (**Supplementary Table S4**). Out of the 52 QTL, 32 were detected by non-nested JLM (significant across the families), 12 by nested JLM (significant within the family), and 8 by MLMM (Multi-locus Mixed Linear Model) (**Supplementary Table S4**). In addition, the QTL detected by non-nested JLM had smaller PVE values compared to the nested JLM (**Supplementary Table S4** & **Supplementary Figure S2B**), since in the nested JLM, marker*family interaction factor causes the inflation of variance component estimation for markers (Schielzeth and Nakagawa, 2013).

### 3.4 Joint linkage analysis for all populations (QTL significant across the populations)

A total of 992 individual samples were retained after the filtration process (missing sites and outliers) and were used for joint linkage association analysis of all populations. The nomenclature used for QTL detected by joint analysis differed from population-specific analysis only for the removal of population names (**Table 3** & **Supplementary Table S5**). The family factor was highly significant for all 6 traits (**Supplementary Table S3**). Again, 1000 permutations were utilized for each trait to correct for multiple testing errors, with final significance thresholds ranging from 2.25E-6 to 1.16E-8. Each identified QTL explained less than 10% of phenotypic variance, further indicating the genetic complexity of these fiber quality traits. Also consistent with single population analysis (**Supplementary Table S4**), the respective subgenomes contributed similar numbers of QTL (8 from the A_t_ subgenome, 11 from the D_t_) (**Supplementary Table S5**).

**Table 3:** Summary of all unique QTL (additive) detected by integrating the results from population-specific analysis and joint analysis of all populations, PVE values were included from joint analysis of all populations except for QTL which were found to be significant only in population-specific analysis where non-nested JLM method’s PVE is shown unless the other two population-specific methods (Non-nested JLM and MLMM) detected the QTL only.

S. No.	QTL_name	Trait	Chromosome	Interval (Mbp)	Position	P-value	PVE	Additive effect	Favorable allele source
1	qMIC_pop2_D01	MIC	D01	29.02-60.17	36997917	1.44E-05	0.123	0.036	DES56
2	qMIC_pop5_D02	MIC	D02	64.13-66.13	64131289	1.73E-05	1.500	0.07	DES56
3	qMIC_A11	MIC	A11	119.07-121.79	120932319	6.17E-16	5.351	0.112	T1046
4	q_MIC_D03	MIC	D03	7.39-41.93	8418950	4.44E-08	2.407	0.088	T1046
5	qMIC_A08	MIC	A08	7.54-78.20	51868732	3.03E-07	2.104	0.069	T326
6	qUHM_D09	UHM	D09	43.03-53.22	43030709	3.23E-08	1.963	0.018	DES56
7	qUHM_pop5_A08	UHM	A08	123.82-125.46	124663792	6.54E-06	0.601	0.011	DES56
8	qUHM_pop4_A13	UHM	A13	12.83-75.30	62146285	9.67E-09	1.643	0.017	DES56
9	qUHM_pop4_A12_1	UHM	A12	107.17-107.57	107178868	3.33E-07	1.272	0.015	T1046
10	qUHM_pop4_A12_2	UHM	A12	6.92-7.09	7094350	1.16E-05	0.919	0.01	T1046
11	qUHM_pop4_D03	UHM	D03	47.99-50.04	49322147	1.85E-06	1.099	0.012	T1046
12	qUHM_D02_1	UHM	D02	4.01-4.56	4526036	5.79E-07	1.599	0.009	T1046,T063
13	qUHM_D02_2	UHM	D02	31.01-41.92	31017663	6.57E-07	1.583	0.007	T1046,T063,T257
14	qUHM_pop2_D01	UHM	D01	58.27-63.18	63189430	3.05E-07	0.289	0.006	T257
15	qUHM_pop1_A08	UHM	A08	25.41-94.14	51171534	1.10E-07	0.786	0.009	T326
16	qUHM_pop1_A03	UHM	A03	52.51-103.71	91727570	8.27E-06	0.538	0.008	T326
17	qUHM_pop1_D06	UHM	D06	6.95-6.95	6957599	1.07E-05	36.033	0.005	T326
18	qUI_A13	UI	A13	12.35-46.67	13507504	4.12E-10	3.204	0.261	DES56
19	qSTR_D01	STR	D01	44.29-61.36	48590025	5.72E-08	1.996	0.262	DES56
20	qSTR_D09	STR	D09	43.03-45.55	45296673	1.32E-07	1.884	0.572	DES56
21	qSTR_pop4_A13	STR	A13	14.69-76.26	48843925	6.71E-06	1.487	0.446	DES56
22	qSTR_pop4_D04	STR	D04	13.78-35.08	13782765	9.50E-06	1.607	0.379 - 0.614	T1046
23	qSTR_D02	STR	D02	31.01-41.92	41641056	1.02E-08	2.227	0.212	T1046, T063, T257
24	qSTR_A07	STR	A07	40.18-44.72	41308176	4.90E-08	2.017	0.841	T326
25	qSTR_pop1_A03	STR	A03	46.89-97.61	59311294	3.46E-06	1.084	0.304	T326
26	qELO_D01_1	ELO	D01	0.44-1.52	1011588	2.96E-10	2.098	0.159	DES56
27	qELO_D11	ELO	D11	64.42-72.91	68450513	6.87E-10	2.009	0.151	DES56
28	qELO_pop2_D08	ELO	D08	56.94-59.47	59475594	4.20E-07	0.197	0.098	DES56
29	qELO_pop6_D05	ELO	D05	32.64-58.06	46813712	2.38E-06	0.198	1.316	DES56
30	qELO_D04	ELO	D04	48.46-53.74	53747393	4.36E-15	3.289	0.108	T1046, T063, T257
31	qELO_A05	ELO	A05	42.37-103.01	71399886	7.27E-09	1.763	0.102	T1046, T063, T257
32	qELO_A01	ELO	A01	25.82-108.47	87693325	4.65E-10	2.051	0.125	T1046, T257
33	qELO_D01_2	ELO	D01	16.94-48.90	44290588	2.73E-10	2.106	0.091	T1046, T281, T063,T326
34	qSFC_A13	SFC	A13	12.35-98.93	13507504	4.23E-11	3.425	0.196	DES56
35	qSFC._pop1_D12	SFC	D12	41.99-48.41	43564507	1.34E-05	0.385	0.121	DES56
36	qSFC_pop4_D04	SFC	D04	21.06-26.37	21067784	9.44E-08	4.283	0.13 - 0.229	T1046
37	qSFC_A01	SFC	A01	69.36-97.18	77901774	4.74E-07	1.979	0.065	T1046, T063, T257, T281
38	qSFC_pop1_D06	SFC	D06	6.95-6.95	6957599	2.74E-06	0.452	0.079	T326

PVE, Percent phenotypic variance explained; Mbp, Million base pairs.

### 3.5 Quantitative trait variation of individual fiber quality traits

Most QTL identified by joint analysis and population-specific analyses (**Supplementary Tables S4, S5**) had overlapping likelihood intervals, thus only increasing the total number of unique QTL from 34 to 38 (**Table 3**).

#### 3.5.1 Micronaire

Among 5 unique QTL for MIC, one or more exotics could improve on the DES56 allele for three. Two QTL (qMIC_A11 and qMIC_A08) were significant within families, across families and populations with cross-population PVE of 5.35% and 2.10% respectively (**Table 3**); and favorable alleles originating from exotic lines (T1046 and T326 respectively) with additive effects (calculated from joint analysis) of 0.112 units and 0.069 units respectively. Another QTL- qMIC_D03, was significant across families in population 4 and across populations with a PVE of 2.40%, the exotic T1046 allele contributing an estimated negative (favorable for MIC usually) additive effect of 0.08 units across populations (**Table 3**). The remaining 2 QTL were significant across families within populations 2 (qMIC_pop2_D01) and 5 (qMIC_pop5_D02), superior alleles originating from DES56 with PVE of 0.12% and 1.5% respectively; and additive effects of 0.035 units and 0.069 units respectively (**Table 3**).

#### 3.5.2 Upper half mean length

Among 12 unique QTL detected for UHM, DES56 improved upon the exotic alleles for 3. Among these 3 QTL- qUHM_D09, qUHM_pop5_A08 and qUHM_pop4_A13, only the first was significant across populations with a PVE of 1.96% (**Table 3**), while the other two QTL were significant across families with PVE values of 0.6% and 1.64% respectively (**Supplementary Table S4**).

Two QTL had multiple sources of favorable exotic alleles, qUHM_D02_1 with two favorable allele sources - T1046 and T063; and qUHM_D02_2 with superior alleles from T1046, T063 and T257 (**Table 3**). The PVE values for these 2 QTL were about 1.59% across the populations with a physical distance of about 27 Mbp between actual associations and non-overlapping intervals (**Table 3**).

A total of 2 QTL- qUHM_pop1_A08 and qUHM_pop5_A08 (mentioned above), were significant in populations 1 and 5 respectively, both located in the A08 chromosome but with non-overlapping genetic regions and a large physical distance between actual associations (about 73 million bps). T326 improved upon the DES56 allele for qUHM_pop1_A08 with a PVE value of 0.78% (**Table 3**). qUHM_pop1_A03 and qUHM_pop1_D06 were significant across the families detected by non-nested JLM and MLMM respectively in population 1. T326 contributed the favorable allele for both QTL (**Table 3**).

Another QTL, qUHM_pop2_D01 was significant both within and across families in population 2 with a PVE value of 0.29% estimated across families and the T257 allele estimated to be superior. Population 4 showed 3 QTL- qUHM_pop4_A12_1, qUHM_pop4_A12_2 and qUHM_pop4_D03, to be significant across families, with favorable alleles for all from T1046 with PVE values of 1.27, 1.09 and 0.91% respectively (**Table 3**).

#### 3.5.3 Uniformity index

As anticipated by the low broad sense heritability in all populations, no QTL was identified for UI by population-specific analysis for any of the 6 populations (**Supplementary Table S4**). However, joint analysis owing to higher statistical power offered by a larger sample size identified qUI_A13, with a p-value of 4.12E-10 and PVE of 3.20% (**Supplementary Table S5**). DES56 improved upon the exotic allele, discouraging introgression from any of the 5 exotic lines tested in this region.

#### 3.5.4 Fiber strength

The total number of unique QTL identified for fiber strength in the present study was 7, with 2 QTL – qSTR_D02 and qSTR_A07, significant within families and across families in populations 4 and 1 respectively. These QTL were also significant across the populations where qSTR_D02 showed T1046, T063 and T257 possessing the favorable allele with a PVE value of 2.01% (**Table 3**). qSTR_A07 was the only QTL that possessed a favorable allele for this trait from T326 in the joint analysis of all populations. Another 2 QTL- qSTR_D01 and qSTR_D09, were significant across the populations with PVE values of 1.99% and 1.88% respectively and discouraging introgression at both loci as DES56 contributed the favorable allele (**Table 3**). Two QTL were identified to be significant across families- qSTR_pop1_A03 and qSTR_pop4_A13, where T326 allele improved upon DES56 and the DES56 allele improved upon T1046 respectively (**Supplementary Table S4**).

#### 3.5.5 Fiber elongation

Owing to its highest broad-sense heritability values in most individual populations and the joint population (**Supplementary Table S6**), ELO yielded the greatest number of QTL both in population-wise analysis and joint analysis, however, most of these QTL were common or had overlapping intervals in both the analyses resulting in total number of unique QTL as 8 (**Table 3**).

Introgression was discouraged at 4 out of these 8 regions- qELO_D01_1, qELO_D11, qELO_pop2_D08, and qELO_pop6_D05, as the DES56 allele was estimated to be contributing the positive additive effects of 0.159, 0.151, 0.098 and 1.316 units along with PVE values of 2.098%, 2.009%, 0.197% and 0.198% respectively (**Table 3**). qELO_D01_1 and qELO_D11 were both significant across populations as well as across families in populations 5 and 4 respectively while qELO_pop2_D08 and qELO_pop6_D05 were significant across families only in populations 2 and 6 respectively.

qELO_D04 was found to be significant across the populations and families in population 2; within the families and across families in population 3 and had overlapping intervals in both the populations and joint analysis (**Supplementary Tables S4, S5**). The PVE value across the populations was calculated as 3.289% and the exotic lines- T1046, T257, and T063 contributed the incremental or favorable allele with an estimated additive effect of 0.108 units (**Table 3**). Another QTL – qELO_D01_2 was significant across the populations and across families in populations 5 and 6 (**Supplementary Tables S4, S5**). The PVE value for this QTL across the populations was 2.106% where the T281, T063, T326, and T1046 allele improved upon that of DES56. Similarly, qELO_A01 was significant across populations, within the families in 2 and 3 populations (**Supplementary Tables S4, S5**), where both the exotic alleles- T1046 and T257 contributed an additive effect of 0.125 units and PVE value of 2.051% across populations (**Table 3**). qELO_A05 was significant across the populations, within families, and across families in population 6, with a PVE value of 1.76% across the populations. T063, T1046 and T257 were estimated to possess the favorable allele for this QTL.

#### 3.5.6 SFC

The number of unique QTL identified was 5. qSFC_A13 was significant across populations and families in population 4 with a PVE value of 3.425%, discouraging introgression since DES56 was estimated to be contributing the favorable allele (**Table 3**, **Supplementary Tables S4, S5**). Another QTL- qSFC_A01 was significant across populations with a PVE of 1.979% and a negative or favorable additive effect of 0.065 units contributed by T1046, T063, T257, and T281. qSFC_pop1_D06 was significant across families in population 1 with a PVE value of 0.45% and a favorable exotic allele contributed by T326. This marker was also significant for UHM as mentioned above, possibly indicating the pleiotropic mode of action for this association. qSFC_pop1_D12 was identified in population 1 with a PVE value of 0.38% and a superior allele from DES56. qSFC_pop4_D04 was significant within families with a PVE (inflation in nested design due to inclusion of interaction factor) (Schielzeth and Nakagawa, 2013) of 4.28% and a T1046 favorable allele (**Supplementary Table S4**).

### 3.6 Dominant QTL

The above analysis using stepwise regression was carried out using the models limited to the additive mode of action. To capture any genomic regions showing dominance effects, we implemented the genotype model in TASSEL v5.2 (Bradbury et al., 2007) without stepwise regression using the same models as for additive QTL analysis. Within population-specific analysis, only a single QTL for SFC was found to be significant in population 4 at a significance threshold of 1E-05 on the D05 chromosome in an interval of 8.98 Mbp – 9.44 Mbp, a dominance effect of -0.304 units and PVE value of 10%. Further, we implemented the same genotype model using the joint analysis of all 6 populations to extract the dominant QTL with a p-value threshold of 1E-07. Only a single association was evident for ELO on chromosome D11, which lacked support as no other markers in its vicinity were significant even at a p-value threshold of 1E-04. The above association detected for SFC in population-specific analysis indicated a p-value of 5.58E-07 in the joint analysis however no adjacent markers were significant at a p-value threshold of 1E-04. Hence, the results from these analyses do not provide strong evidence for genomic regions exhibiting dominance effects for these 6 fiber quality traits.

### 3.7 Contribution of DES56 or elite line/genomic regions need to be preserved

**Figure 5:**
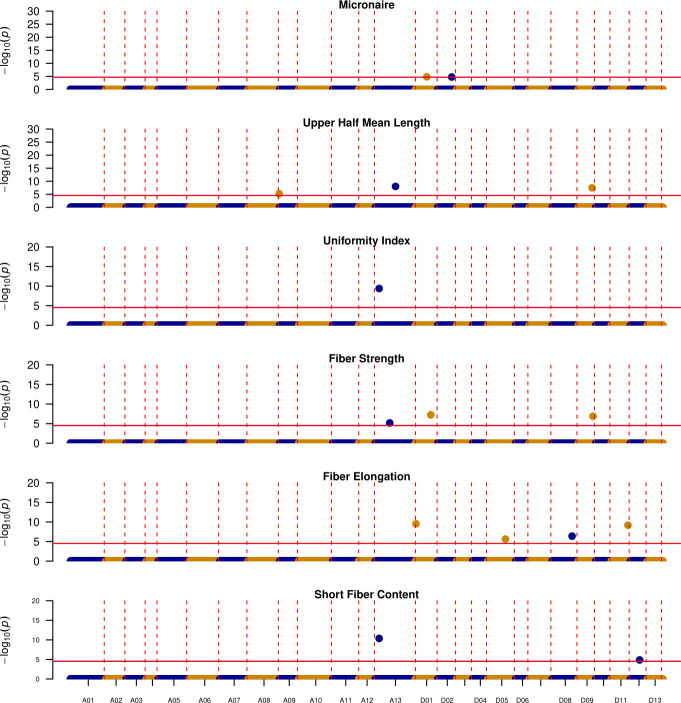
Manhattan plots depicting the location of QTL where DES56 or elite parent is contributing the favorable allele, Vertical red dotted lines indicate the boundaries for each chromosome (x-axis), while horizontal solid red lines indicate the significance threshold ~ 1E-5, y-axis denotes the -log_10_(p-value) of each association.

**Figure 6:**
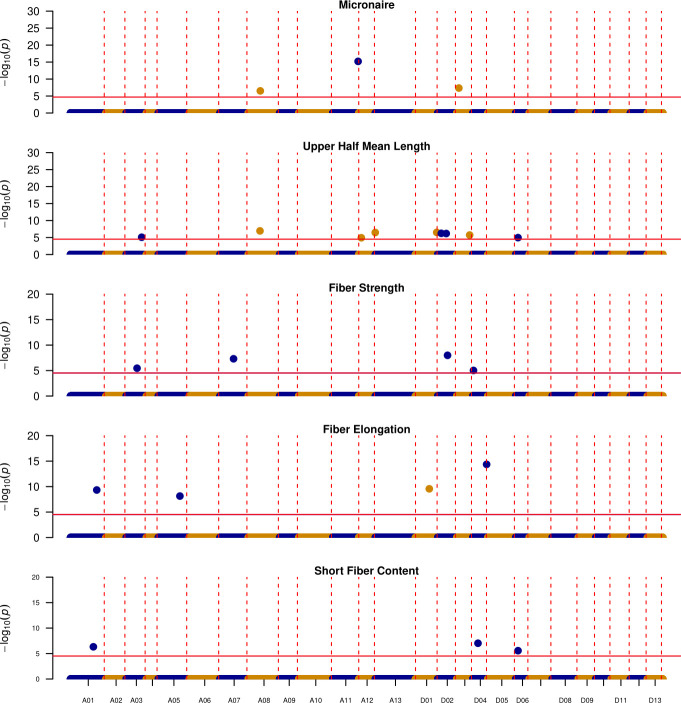
Manhattan plots depicting the location of QTL where at least one of the exotic lines is contributing the favorable allele, Vertical red dotted lines indicate the boundaries for each chromosome (y-axis), while horizontal solid red lines indicate the significance threshold ~1E-5, y-axis denotes the -log_10_(p-value) of each association.

Overall, out of 38 unique QTL, 15 discourage introgression from these exotic lines as DES56 confers favorable alleles, with 5 located in the A_t_ and 10 in the D_t_ subgenome (**Figure 5**). Of these 15 genomic regions, chromosome A13 harbors 4, affecting UHM, UI, STR, and SFC. These genomic regions start at almost 12 Mbp and end at about 98 Mbp for SFC while for other traits intervals are relatively smaller ending almost at 75 Mbp (**Table 3**). Similarly, the genomic region located on chromosome D09 from 43.03 Mbp -53.22 Mbp harbors favorable DES56 alleles for UHM and STR, with no favorable alleles from these exotic lines (**Table 3**; **Figure 5**). Chromosome D01 presents a more complex pattern where 3 QTL affecting 3 traits- MIC, STR and ELO (0.44 Mbp-61.36 Mbp) have favorable DES56 alleles while 2 QTL affecting UHM and ELO (16.94 Mbp -63.18 Mbp) had superior alleles originating from T257 for UHM and from T1046, T281, T063 for ELO (**Table 3**, **Figures 5**, **6**). Apart from these regions, there were other QTL regions listed in **Table 3** and **Figure 5**.

### 3.8 Contribution of exotic lines

For the other 23 QTL regions, exotic alleles improved upon DES56 (**Table 3**, **Figure 6**). Out of these 23 QTL, T1046 (exotic parent in populations 3 and 4) contributed the favorable alleles for 15 QTL (**Table 3**). Of these 15 genomic regions, 9 originated from the D_t_ subgenome and UHM had the greatest number of QTL (5) followed by ELO (4) and STR (2) respectively. D02 is of particular importance as it possessed 3 QTL – two affecting UHM and one affecting ELO (**Table 3**; **Figure 6**). Similarly, A12 possessed 2 QTL regions that had positive estimated effects of the T1046 allele for UHM. Chromosome D03 harbors two QTL regions possibly contributing superior MIC and UHM values in T1046 as compared to DES56. Moreover, another QTL region (119.07 Mbp -121.79 Mbp) located on the A11 chromosome could potentially contribute superior MIC values if introgressed with the T1046 allele. Three QTL were identified on the D04 chromosome affecting ELO, STR, and SFC. However, the QTL- qELO_D04 (48.46 Mbp -53.74 Mbp) affecting ELO was the only QTL that was significant across populations while the other two were significant only within the families in population 4 (**Supplementary Tables S3, S5**).

Some regions indicated multiple exotic lines possessing the same favorable allele (**Table 3**). Two QTL controlling SFC and ELO had overlapping regions on chromosome A01 where both T1046 and T257 improved upon the DES56 allele (**Table 3**). At least two of three lines T1046, T063 and T257 possessed the same allele that could be introgressed into the DES56 background for 8 QTL regions affecting 4 fiber quality traits (**Table 3**). Two such regions belong to D02 affecting the UHM as mentioned above. 2 QTL regions – D01(16.94 Mbp -48.90 Mbp) and D04 (48.46 Mbp -53.74 Mbp) were affecting ELO where these three exotic lines share the same favorable allele (**Table 3**). These QTL regions hold more confidence along with the multiple options for introgression since they share the same favorable allele among multiple populations.

T326 can potentially serve as the introgression line for 8 genomic regions where 2 QTL regions are located on the A08 chromosome with overlapping regions affecting MIC and UHM. Two overlapping QTL regions on chromosome A03 improved upon the DES56 allele for STR and UHM along with a QTL region on D01 for ELO, where 3 other lines also possessed the same positive allele (**Table 3**).

## 4 Discussion

### 4.1 Use of diverse parents and their contribution

If we consider all 5 exotic lines jointly representing the broad spectrum of the exotic *G. hirsutum* gene pool, exotic alleles that improve on those of DES56 are rare or absent at most loci. In contrast, at a small minority of loci (8 in this study), the elite gene pool may be inherently poor (where multiple exotic lines possess favorable alleles). For 15 of the 38 QTL detected, none of the 5 exotic alleles improved on the DES56 allele while for another 15, only one among the 5 exotic alleles improved on DES56 (3 unique alleles from each exotic line for 30 comparisons with DES56). Thus for 150 comparisons, we can speculate that up to 15 rare favorable alleles may have been ‘left behind’ in domestication for each exotic line, presumably not sampled in the potentially small number of cottons from which the elite gene pool was formed (Lubbers and Chee, 2009). For the remaining 8 QTL, we can speculate that in about 13 out of 40 comparisons, multiple exotic alleles improved upon the DES56 allele.

These 8 QTL reveal much about the elite gene pool and opportunities for its improvement. At five of these loci, T1046, T063, and T257 all contributed alleles that improved on the DES56 allele, consistent with the genome-wide patterns of relatedness shown by the PCA plot (**Figure 1**). Collectively, T1046 contributed favorable alleles for 15 QTL regions which was the highest among 5 exotic lines used in the present study. While the estimated genetic effects for this line were unfavorable for fiber length and elongation in a previous study (McCarty et al., 2007), five and four QTL regions detected in the present study suggested this line to possess favorable alleles for these traits respectively. T281 was found to have the highest pairwise genetic distance with DES56. However, only two QTL were identified where this line improved over the DES56 allele. This suggests that the most promising exotic sources of beneficial diversity for fiber quality traits in the current study could be T1046, T063, and T326.

### 4.2 Similarity among the lines

The principal component analysis (**Figure 1**) and the pairwise genetic distance calculations (**Table 1**) revealed that two of the five lines are relatively closely related to DES56. Additionally, each exotic line exhibited a higher genetic similarity to the common line DES56 compared to the other exotic lines, a finding consistent with previous studies (Liu et al., 2000). Although, this high relative relatedness could be attributed to the possible evolutionary or domestication events that may have caused the elite gene pool to accumulate the beneficial alleles from all of these exotic lines, however, it could also possibly be the result of the retention of extra chromatin in these race stocks during the repeated backcrossing or conversion process (McCarty et al., 1979). This highlights the significance of using DNA marker-assisted introgression to recover a maximum of the recurrent genetic background (Liu et al., 2000). Moreover, we advocate comparative genomic analysis of the original primitive accessions with the converted ones to identify and measure the actual gap.

### 4.3 Number of QTL detected and benefit of using multiple approaches

Looking at the large proportion of variance due to the environment, we predicted the breeding values constituting mostly genetic effects. This helps to detect QTL which perform stably in the face of extremely different environments.

Population-specific analysis identified 34 unique QTL for 6 fiber quality traits while the joint analysis identified 19 QTL, where most of the QTL (15) were common across the methods making it to the final number of unique QTL (QTL common to both approaches are counted once) identified in this study to 38. Population-specific analyses help to extract the QTL significant within a population since the joint analysis does not target the sites where exotic lines introduce more than one type of allele (restricting to only biallelic sites) opposite to the reference or DES56 allele. On the other hand, joint analysis of all populations, owing to the increased sample sizes helps with the dissection of associations that can’t be extracted with smaller sample sizes along with more precise estimations of QTL parameters. In the present study, 4 such associations were extracted by joint analysis which were not significant in population-specific analyses. In addition to that, UI, owing to its lower genetic variation yielded only a single QTL significant across populations contrary to no QTL being capable of surpassing the permutation-based significance threshold in the population-specific analysis, possibly indicating the higher power of these joint linkage association analysis models (Myles et al., 2009).

### 4.4 Subgenomic distribution of QTL

Interestingly, 16 QTL out of 38 were located in the A_t_ subgenome while 22 originated from the D_t_ subgenome, as has been evident in various other QTL mapping studies (Adhikari et al., 2017; Kumar et al., 2019) and meta-QTL studies (Rong et al., 2007; Xu et al., 2020). Since diploid cotton species with the D genome such as *G. raimondii* and *G. gossypioides* do not produce spinnable fiber, these findings suggest the possibility of some evolutionary mechanisms like subgenomic exchange or possibly the activation of certain genomic regions after polyploidization (Jiang et al., 1998; Paterson et al., 2012). Another possible explanation could be the lack of certain genomic regions in diploid D genome species which are crucial for the complete development of fiber cells, while the variation within the other regions involved in various stages of fiber development process could contribute to the QTL being identified in the D_t_ subgenome in allotetraploid cotton. Future, comparative genomics and evolutionary studies might provide more clear insights into these possibilities.

Moreover, A02, A04, A06, A09, and A10 stayed QTL-less in the A_t_ subgenome while in the D_t_ subgenome, D07, D10, and D13 didn’t show any genomic region affecting any of these 6 fiber quality traits. A recent meta-analysis also suggested A04 and D13 to be mostly devoid of major meta-QTL regions for these fiber quality traits (Xu et al., 2020).

### 4.5 Small-effect QTL in majority

Nested JLM and MLMM were included in the study to confirm the QTL regions which are significantly identified by multiple methods to generate more confidence, however, PVE and QTL effects from non-nested JLM and joint analysis of all populations were considered mostly, because the nested JLM model yields inflated PVE values due to the confounding of family*marker interaction variance components with the marker variance component (Schielzeth and Nakagawa, 2013), and MLMM performs too conservatively in the populations with controlled mating designs, allocating most of the genetic background effects to the kinship elements (Hoffman, 2013), and inflating the estimated variance for detected QTL due to the differences in handling of background variance as compared to the stepwise regression in TASSELv5.2 (Bradbury et al., 2007).

All QTL detected were small effects or minor QTL with PVE values of less than 10%. The prevalence of these small-effect QTL strengthens the concept of the genetic complexity of inheritance for these fiber quality traits as reported in previous studies (Chandnani et al., 2018). However, QTL with >1.5% PVE value for joint analysis of all populations (~1000 sample size) should be of high importance since this parameter is greatly inflated by a decrease in sample size from thousands to hundreds (Beavis, 1995). In particular, a QTL detected for MIC on chromosome A11 had a PVE value of 5.3% with a negative additive effect of 0.112 units for T1046 (**Table 3**), which could significantly improve this trait if employed in introgressive breeding, controlling for linkage drag effects if any.

### 4.6 Pleiotropic or multi-trait QTL-

There were several associations common or with overlapping QTL intervals among the 6 fiber quality traits reflecting the possible pleiotropic mode of action for a single QTL region (Scholl and Miller, 1976) or tightly linked multiple QTL (Meredith, 1977). In particular, UHM and STR have shown 5 such potential QTL intervals on A03, A13, D01, D02, and D03 (Table 4), the correlation analysis among these two traits suggests a strong positive correlation (**Figure 4**) which is also reported in other QTL mapping studies (Ademe et al., 2017; Adhikari et al., 2017; Kumar et al., 2019). Chromosome D01 had such a QTL region possibly affecting STR and ELO, along with a significant negative correlation among these two traits indicating the possible action of two linked QTL such that an incremental allele for one trait will decrease the phenotypic value of another trait. This region is particularly important since UHM and MIC also share an overlapping region in which a significant negative correlation indicates a similar dual mode of action. Similarly, A08 possessed two significant associations for MIC and UHM within 1 Mbp of physical distance along with overlapping intervals where a significant negative correlation indicates the possible pleiotropic mode of action. The only QTL identified for UI also affected SFC. Such a genomic region affecting two traits – STR and SFC, found significant associations about 8 Mbps apart on the D04 chromosome. These genetic regions could be of great significance, commercially assisting with the improvement of multiple traits at the same time.

### 4.7 Congruence with previous studies and novel QTL

Since we utilized the TM-1 reference genome sequence, we referred to meta-QTL analysis performed on studies that used the same genotype as the reference (Xu et al., 2020). An important QTL found on the lower end of chromosome A11 (119.07-121.79 Mbp) (Table 4) is close to a meta-QTL identified for MIC (Xu et al., 2020). Similarly, a QTL interval identified on chromosome D03 coincides with a meta-QTL (meta-QTL-68 in Xu et al., 2020). Chromosome D09 and D02 harbored two QTL (qUHM_D09 and qUHM_D02_1 respectively) which coincides largely with the only meta-QTL identified for UHM or fiber length in these chromosomes (Table 4) (Xu et al., 2020). An association found on D06 for UHM at 6957599 falls within the only meta-QTL identified on this chromosome (5.35-17.71 Mbp in XU et al., 2020). Interestingly this association was also found to be significant for STR (Table 4). qSTR_pop1_A03 had the overlapping interval with the only meta-QTL identified on A03 for fiber strength. qELO_D04 was identified close to the only meta-QTL identified on this chromosome (40.11-48.77 Mbp in Xu et al., 2020) for ELO. Another QTL- qELO_D01_2 coincided with one of the meta-QTL identified on the D01 chromosome for ELO (Xu et al., 2020). Along with these QTL which coincide or are located close to the verified meta-QTL, the other novel QTL generate opportunities for future meta-analysis studies, where the regions that are not yet confirmed to acquire the meta-QTL status could possibly gain more confidence to emerge as the new important consensus regions for these fiber quality traits.

### 4.8 Lower genetic variation for uniformity index

As has been reported in various other studies, heritability values for the uniformity index are comparatively lower than for other fiber quality traits (Ademe et al., 2017; Adhikari et al., 2017; Kumar et al., 2019), which suggests using powerful association techniques and large sample sizes (preferably 1000 or more) along with multi-environmental phenotyping to extract these genetic regions precisely. This is evident in the present study where population-specific analysis could not identify any genomic regions due to its lower sample size (less than 200), while joint analysis identified a QTL on the A13 chromosome with a PVE value of 3.20% across the populations. However, there were about 11 families across 24 studied that showed superior transgressive mean values for the predicted breeding values, indicating the possibility of multiple small effect allelic combinations complementing each other’s effect, as is generally considered in the case of complex quantitative traits (Mackay et al., 2009; Paterson et al., 2012). Hence, particularly for this trait, genomic selection techniques could potentially complement marker-assisted selection techniques in the breeding operations, where the former takes into account the total genetic variation at the whole genome level rather than targeting a certain genetic region as in the latter (Islam et al., 2020; Li et al., 2022).

### 4.9 Implications in cotton breeding

The results from the present study and previous studies (McCarty et al., 1996, 2007) motivate the utilization of these exotic lines, especially T1046, T063 and T326, in various fiber quality improvement breeding programs. Moreover, the results indicated these lines particularly contribute alleles that could target multiple traits simultaneously. In addition to that, UHM and STR have shown a strong positive correlation among each other and multiple QTL affecting both traits, motivating the utilization of such QTL regions. Although the uniformity index had lower genetic variation, the positive transgressive values indicate these lines’ potential for improving this trait. ELO had the highest proportion of genetic variation, along with half of the total QTL detected for this trait indicating multiple exotic alleles as the favorable ones. Hence, this particular trait also presents a high opportunity for improvement by the utilization of this exotic gene pool. The QTL identified within the established meta-QTL regions strengthen their credibility in marker-assisted selection. The results from our study and numerous previous studies (Ademe et al., 2017; Adhikari et al., 2017; Chandnani et al., 2018; Chee P. et al., 2005; Draye et al., 2005) have generated enough evidence about the complexity of these fiber quality traits such that multiple genomic regions with small effects complement each other’s effects or regulate coordinately to contribute to the total genetic variation (Paterson et al., 2012). This motivates the implementation of genomic selection techniques which will help with the utilization of cumulative effects of most of these QTL distributed genome-wide (Islam et al., 2020; Li et al., 2022).

## Data Availability

Supplementary files relating to original contributions have been uploaded in the supplementary section (Data Sheet 1.docx). The raw sequenced files for each population have been deposited in NCBI SRA bio project number PRJNA1215963. The phenotypic data and sample barcode information for the sequenced files have been uploaded to the supplementary section (Table 1.xlsx). The URL for NCBI SRA bio project accessions is https://www.ncbi.nlm.nih.gov/sra/?term=PRJNA1215963.
