# Supplementary material for: Identification of favorable alleles from exotic Upland cotton lines for fiber quality improvement using multiple association models
Source: Front Plant Sci. 2025 Apr 16;16:1553514. doi: 10.3389/fpls.2025.1553514 (PMC12042663; doi:10.3389/fpls.2025.1553514)
Supplement: Supplementary file 1 [file DataSheet1.docx]

***Supplementary Material***

1. ***Supplementary Tables***

**Supplementary Table 1A-** Table depicting the botanical race identity and country of origin for the exotic parents used in the day-neutral conversion breeding program.

| Parent name | Race | Country |
| --- | --- | --- |
| T326 | Latifolium | * |
| T1046 | Yucatanense | Mexico |
| T257 | Morrillii | Mexico |
| T281 | * | * |
| T063 | Latifolium | Mexico |

**“*”** indicates no information available

**Supplementary Table 1B-** Table depicting the phenotypic values recorded for 6 fiber quality traits for 6 parental lines used in the present study during 2012 and 2013.

| line | Environment | MIC | UHM | UI | STR | ELO | SFC |
| --- | --- | --- | --- | --- | --- | --- | --- |
| DES56 | 2012 | 4.74 | 1.1 | 83.8 | 29.1 | 4.8 | 7.5 |
| DES56 | 2012 | 4.55 | 1.09 | 82 | 28.9 | 5 | 7.9 |
| DES56 | 2012 | 5.26 | 1.08 | 81.3 | 28.9 | 5.2 | 8.5 |
| DES56 | 2012 | 4.82 | 1.09 | 81.4 | 28 | 5 | 8.4 |
| DES56 | 2012 | 4.52 | 1.12 | 83.3 | 29.7 | 5 | 7.7 |
| T063 | 2012 | 3.38 | 1.19 | 82.7 | 32.8 | 5.4 | 7.8 |
| T063 | 2012 | 4.34 | 1.14 | 82.5 | 30.6 | 6 | 7.9 |
| T257 | 2012 | 5.57 | 1.03 | 82.5 | 29.4 | 5.3 | 7.5 |
| T257 | 2012 | 4.43 | 1.04 | 81.4 | 28.2 | 5.3 | 7.4 |
| T257 | 2012 | 4.59 | 1.17 | 84.6 | 30.7 | 4.7 | 6.8 |
| T281 | 2012 | 5.55 | 1.01 | 82.8 | 28.3 | 6.8 | 7.1 |
| T281 | 2012 | 5.61 | 0.9 | 81.1 | 26.5 | 7.3 | 6.8 |
| T281 | 2012 | 5.38 | 0.94 | 81.6 | 27.2 | 6.5 | 7.6 |
| T281 | 2012 | 5.7 | 0.88 | 81.2 | 28.8 | 7.6 | 7.9 |
| T281 | 2012 | 5.45 | 0.88 | 80.2 | 27.8 | 8.8 | 8.5 |
| T281 | 2012 | 5.7 | 0.85 | 78.8 | 31.4 | 9.2 | 9.7 |
| T281 | 2012 | 5.3 | 0.86 | 78 | 29.7 | 9 | 12 |
| T326 | 2012 | 3.67 | 0.92 | 81.4 | 23.9 | 6.3 | 10.7 |
| T1046 | 2012 | 4.4 | 1.09 | 83.6 | 31.7 | 4.9 | 7.4 |
| T1046 | 2012 | 4.5 | 1.07 | 83 | 28.3 | 7.5 | 7.2 |
| T1046 | 2012 | 3.91 | 1.15 | 84.9 | 29.7 | 7.9 | 6.6 |
| T1046 | 2012 | 3.09 | 1.05 | 79.8 | 30.2 | 7.8 | 10.4 |
| T1046 | 2012 | 4.66 | 1.09 | 83.2 | 29.9 | 5.7 | 7.3 |
| T1046 | 2012 | 3.49 | 1.11 | 84.3 | 30.7 | 5.4 | 7 |
| DES56 | 2012 | 4.4 | 1.1 | 80.9 | 29.6 | 4.7 | 8.7 |
| DES56 | 2012 | 4.34 | 1.06 | 81.9 | 27.3 | 5.3 | 8.5 |
| DES56 | 2012 | 4.48 | 1.06 | 81.6 | 28.1 | 5.1 | 8.4 |
| DES56 | 2012 | 4.79 | 1.08 | 82.3 | 28 | 5 | 8.2 |
| T063 | 2012 | 3.79 | 1.25 | 85.1 | 31.8 | 7.5 | 7.2 |
| T063 | 2012 | 3.11 | 1.17 | 83.4 | 30.2 | 6.9 | 7.8 |
| T063 | 2012 | 2.96 | 1.19 | 82.9 | 34.2 | 7.3 | 8.2 |
| T063 | 2012 | 3.75 | 1.1 | 82.1 | 27.3 | 7.1 | 8.7 |
| T063 | 2012 | 3.74 | 1.23 | 83.1 | 29.9 | 6.3 | 8.1 |
| T257 | 2012 | 4.96 | 1.02 | 82.7 | 30 | 6.4 | 7.2 |
| T257 | 2012 | 4.7 | 1.07 | 83.4 | 30.1 | 6.1 | 6.9 |
| T257 | 2012 | 4.9 | 1.07 | 83.2 | 30.5 | 6.9 | 6.2 |
| T281 | 2012 | 5.37 | 0.88 | 80.3 | 25.9 | 9.2 | 8.3 |
| T281 | 2012 | 5.73 | 0.86 | 79.2 | 26.6 | 8.8 | 9.6 |
| T281 | 2012 | 5.58 | 0.86 | 79.6 | 26.9 | 8.8 | 9.5 |
| T281 | 2012 | 5.02 | 0.9 | 81.4 | 26.3 | 8.6 | 7.3 |
| T281 | 2012 | 4.89 | 0.89 | 79.9 | 27.4 | 8.8 | 11.5 |
| T281 | 2012 | 5.03 | 0.89 | 78.5 | 25.9 | 8.9 | 9.4 |
| T281 | 2012 | 4.96 | 0.9 | 79.6 | 28.8 | 9.3 | 7.7 |
| T326 | 2012 | 3.35 | 0.86 | 77.2 | 25.4 | 7.6 | 13.8 |
| T1046 | 2012 | 4.98 | 0.99 | 80.2 | 28.3 | 6.4 | 9.2 |
| T1046 | 2012 | 4.58 | 1.08 | 83.8 | 27.7 | 7.6 | 7.7 |
| T1046 | 2012 | 5.38 | 1.09 | 83.1 | 28.7 | 6.2 | 6.8 |
| T1046 | 2012 | 4.39 | 1.15 | 84.5 | 31.1 | 5.9 | 6.7 |
| T1046 | 2012 | 4.56 | 1.23 | 84.4 | 33 | 5.9 | 7.6 |
| DES56 | 2013 | 4.78 | 1.05 | 81.8 | 26.1 | 6.3 | 10.3 |
| DES56 | 2013 | 4.38 | 1.07 | 79.8 | 26.6 | 5.4 | 10.5 |
| DES56 | 2013 | 4.93 | 1.09 | 81.9 | 27.2 | 5.1 | 8.7 |
| DES56 | 2013 | 4.41 | 1.03 | 80.9 | 25.7 | 5.4 | 9.4 |
| DES56 | 2013 | 4.72 | 1.03 | 82.3 | 25 | 5.4 | 9.3 |
| T063 | 2013 | 3.86 | 1.15 | 84.2 | 27.3 | 7.1 | 7.7 |
| T063 | 2013 | 3.99 | 1.13 | 82.6 | 26.8 | 5.7 | 8.9 |
| T063 | 2013 | 4.09 | 1.1 | 83.1 | 27.5 | 6.8 | 8.4 |
| T063 | 2013 | 3.43 | 1.12 | 82.9 | 28.5 | 5.8 | 8.6 |
| T257 | 2013 | 4.85 | 0.96 | 80.8 | 26.4 | 4.7 | 8.5 |
| T257 | 2013 | 4.74 | 1.03 | 83.8 | 30.1 | 4.6 | 6.6 |
| T257 | 2013 | 4.28 | 1.02 | 82 | 25.8 | 5.3 | 8.3 |
| T281 | 2013 | 5.46 | 0.92 | 79.4 | 27.7 | 6.9 | 10.8 |
| T281 | 2013 | 5.71 | 0.94 | 79.8 | 24 | 6.9 | 9.9 |
| T281 | 2013 | 5.58 | 0.9 | 78.9 | 27.2 | 7 | 11.1 |
| T281 | 2013 | 5.51 | 0.84 | 77.6 | 27 | 7.1 | 14.1 |
| T281 | 2013 | 5.87 | 0.94 | 80 | 24.1 | 6.6 | 9.1 |
| T281 | 2013 | 4.85 | 0.89 | 78.4 | 26.5 | 7.3 | 11.1 |
| T281 | 2013 | 5.14 | 1.04 | 81.3 | 25.1 | 6 | 8.9 |
| T281 | 2013 | 5.26 | 0.99 | 82.7 | 25.8 | 6.4 | 7.9 |
| T281 | 2013 | 5.61 | 0.95 | 81.5 | 25.4 | 6.2 | 8.4 |
| T281 | 2013 | 5.29 | 0.97 | 79.3 | 26.7 | 7.4 | 10.2 |
| T326 | 2013 | 4.15 | 0.86 | 78 | 22.6 | 7.1 | 13.9 |
| T326 | 2013 | 4.46 | 0.92 | 77.4 | 24.5 | 7 | 12.1 |
| T326 | 2013 | 4.78 | 0.91 | 75.1 | 23.1 | 7.1 | 14.3 |
| T326 | 2013 | 3.26 | 0.91 | 79.6 | 22.3 | 6.5 | 12.9 |
| T1046 | 2013 | 4.45 | 0.95 | 81.2 | 25 | 8.4 | 9.7 |
| T1046 | 2013 | 3.76 | 1.04 | 82.8 | 29.1 | 6.4 | 7 |
| T1046 | 2013 | 4.55 | 1.09 | 82.3 | 30.2 | 5.6 | 8.4 |
| T1046 | 2013 | 4.15 | 1.08 | 82.9 | 28 | 5.8 | 8.1 |
| T1046 | 2013 | 4.7 | 1.01 | 82.5 | 25.2 | 5.4 | 8 |
| T1046 | 2013 | 5.2 | 1.1 | 82.8 | 26.8 | 5 | 8.7 |
| DES56 | 2013 | 4.78 | 1.06 | 81.5 | 27 | 6.1 | 9.3 |
| DES56 | 2013 | 4.82 | 1.09 | 83.5 | 26.4 | 5.5 | 8.6 |
| DES56 | 2013 | 4.5 | 1.1 | 82.6 | 27.6 | 5.3 | 8.2 |
| DES56 | 2013 | 4.57 | 1.02 | 80.1 | 26.2 | 6 | 9 |
| DES56 | 2013 | 5.29 | 1.03 | 80.2 | 23.7 | 5 | 10 |
| T063 | 2013 | 3.92 | 1.12 | 83.6 | 28.7 | 8.7 | 7.1 |
| T063 | 2013 | 3.54 | 1.11 | 83.2 | 29.4 | 8.3 | 7.5 |
| T063 | 2013 | 4.07 | 1.13 | 83.5 | 28 | 7.4 | 7.6 |
| T063 | 2013 | 4.55 | 1.07 | 81.1 | 27 | 7.3 | 8.5 |
| T063 | 2013 | 4.62 | 1.06 | 83.3 | 26.7 | 6.8 | 7.6 |
| T063 | 2013 | 4.65 | 1.06 | 81.7 | 26.9 | 8.2 | 9.1 |
| T257 | 2013 | 4.31 | 1.02 | 82.1 | 26.1 | 6.3 | 7.8 |
| T257 | 2013 | 4.8 | 0.95 | 82.4 | 26.2 | 8 | 7.7 |
| T257 | 2013 | 4.88 | 1.04 | 80.8 | 28.1 | 6.2 | 8.4 |
| T257 | 2013 | 4.98 | 1.06 | 80.9 | 25.3 | 6.1 | 8.9 |
| T257 | 2013 | 4.55 | 1.11 | 83.7 | 29.8 | 7.4 | 6.8 |
| T281 | 2013 | 4.73 | 0.96 | 81.6 | 24.3 | 6.3 | 7.4 |
| T281 | 2013 | 5.33 | 0.89 | 79.3 | 24.8 | 7.3 | 10.1 |
| T281 | 2013 | 5.42 | 0.91 | 81.6 | 23.6 | 7.4 | 9 |
| T281 | 2013 | 5.72 | 0.87 | 77.9 | 25.9 | 8 | 10.8 |
| T281 | 2013 | 5.25 | 0.93 | 81.7 | 26.4 | 6.4 | 7.8 |
| T281 | 2013 | 5.14 | 0.95 | 78.7 | 25.6 | 7.1 | 10.2 |
| T281 | 2013 | 5.06 | 0.97 | 80.7 | 26.8 | 7.2 | 7.6 |
| T281 | 2013 | 5.02 | 0.98 | 82.6 | 26.6 | 8.4 | 7.9 |
| T281 | 2013 | 5.41 | 1.03 | 81.5 | 27.1 | 7.2 | 9.3 |
| T281 | 2013 | 5.55 | 0.91 | 81.9 | 24.9 | 8.7 | 7.7 |
| T326 | 2013 | 3.04 | 0.99 | 82.7 | 24 | 6.2 | 9.1 |
| T326 | 2013 | 4.69 | 0.95 | 82.4 | 25.1 | 8.2 | 6.3 |
| T326 | 2013 | 4.93 | 0.9 | 80.3 | 24.9 | 8.3 | 10.2 |
| T326 | 2013 | 4.34 | 0.91 | 79.8 | 26.2 | 7.2 | 8.2 |
| T326 | 2013 | 4.39 | 0.96 | 79.7 | 25.1 | 8 | 9.2 |
| T1046 | 2013 | 4.8 | 1.06 | 82.2 | 28.5 | 6.5 | 8.7 |
| T1046 | 2013 | 4.51 | 0.98 | 83.7 | 23.8 | 6.6 | 7.6 |
| T1046 | 2013 | 4.81 | 1.02 | 81 | 26.5 | 7.4 | 8.2 |
| T1046 | 2013 | 4.55 | 0.99 | 80.3 | 24.7 | 7.5 | 10.5 |
| T1046 | 2013 | 4.45 | 1.12 | 84.7 | 29.4 | 7.2 | 6.5 |
| T1046 | 2013 | 4.13 | 1.13 | 84.2 | 30.5 | 6.4 | 7.9 |
| T1046 | 2013 | 4.03 | 1.07 | 81.9 | 26.4 | 6.3 | 8.3 |
| T1046 | 2013 | 4.58 | 1.1 | 82.9 | 29 | 5.6 | 7.9 |

**Supplementary Table 1C-** ANOVA table depicting the significance of parental line (genotype of each parental line) for each of the 6 fiber quality traits shown in Supplementary table 1b.

| **Source** | **Df** | **Sum_Sq** | **Mean_Sq** | **F_value** | **Pr_F** | **Trait** |
| --- | --- | --- | --- | --- | --- | --- |
| Parent | 5 | 32.20407 | 6.440813 | 36.92365 | 2.40E-22 | MIC |
| Residual | 114 | 19.8857 | 0.174436 | NA | NA | MIC |
| Parent | 5 | 0.814862 | 0.162972 | 62.22049 | 5.55E-31 | UHM |
| Residual | 114 | 0.298597 | 0.002619 | NA | NA | UHM |
| Parent | 5 | 194.3472 | 38.86944 | 19.33965 | 6.68E-14 | UI |
| Residual | 114 | 229.1208 | 2.009831 | NA | NA | UI |
| Parent | 5 | 218.7524 | 43.75048 | 12.05973 | 2.21E-09 | STR |
| Residual | 114 | 413.5709 | 3.627815 | NA | NA | STR |
| Parent | 5 | 80.64605 | 16.12921 | 19.5839 | 4.85E-14 | ELO |
| Residual | 114 | 93.88987 | 0.823595 | NA | NA | ELO |
| Parent | 5 | 105.4724 | 21.09449 | 11.24526 | 7.95E-09 | SFC. |
| Residual | 114 | 213.8475 | 1.875855 | NA | NA | SFC. |

Sum_Sq = Sum of squares, Df = degrees of freedom, Mean_Sq = Mean sum of squares

**Supplementary Table 2-** ANOVA analysis depicting the significance of genotype and environment for 6 fiber quality traits in each population

| **Df** | **Sum Sq** | **Mean Sq** | **F value** | **Pr(>F)** | **Trait** | **Predictor** | **Population** |
| --- | --- | --- | --- | --- | --- | --- | --- |
| 2 | 65.88123238 | 32.94061619 | 271.263989 | 4.29E-70 | MIC | environment | 1 |
| 168 | 44.68892028 | 0.266005478 | 2.19053908 | 8.48E-10 | MIC | genotype | 1 |
| 326 | 39.5874179 | 0.121433797 | NA | NA | MIC | Residuals | 1 |
| 2 | 0.293862203 | 0.146931102 | 108.586211 | 6.85E-37 | UHM | environment | 1 |
| 168 | 0.587656537 | 0.003497956 | 2.58508741 | 1.22E-13 | UHM | genotype | 1 |
| 327 | 0.442473035 | 0.001353129 | NA | NA | UHM | Residuals | 1 |
| 2 | 46.14973132 | 23.07486566 | 15.444064 | 3.89E-07 | UI | environment | 1 |
| 168 | 298.6180698 | 1.777488511 | 1.18967741 | 0.093590757 | UI | genotype | 1 |
| 328 | 490.0624559 | 1.494092853 | NA | NA | UI | Residuals | 1 |
| 2 | 812.1803155 | 406.0901578 | 201.822199 | 7.21E-58 | STR | environment | 1 |
| 168 | 860.5299112 | 5.122201852 | 2.54567618 | 2.84E-13 | STR | genotype | 1 |
| 328 | 659.9748315 | 2.012118389 | NA | NA | STR | Residuals | 1 |
| 2 | 93.54160593 | 46.77080296 | 259.357268 | 2.85E-68 | ELO | environment | 1 |
| 168 | 58.15369831 | 0.346152966 | 1.91951564 | 2.83E-07 | ELO | genotype | 1 |
| 328 | 59.14938684 | 0.180333496 | NA | NA | ELO | Residuals | 1 |
| 2 | 56.24908746 | 28.12454373 | 34.2030436 | 3.25E-14 | SFC | environment | 1 |
| 168 | 186.5116454 | 1.110188365 | 1.3501311 | 0.011280914 | SFC | genotype | 1 |
| 327 | 268.886182 | 0.822281902 | NA | NA | SFC | Residuals | 1 |
| 2 | 65.62613274 | 32.81306637 | 240.755335 | 1.29E-66 | MIC | environment | 2 |
| 179 | 39.49429522 | 0.220638521 | 1.61886428 | 7.15E-05 | MIC | genotype | 2 |
| 352 | 47.97484281 | 0.136292167 | NA | NA | MIC | Residuals | 2 |
| 2 | 0.229103498 | 0.114551749 | 102.812892 | 6.84E-36 | UHM | environment | 2 |
| 179 | 0.405797816 | 0.002267027 | 2.03471003 | 8.43E-09 | UHM | genotype | 2 |
| 352 | 0.392190268 | 0.001114177 | NA | NA | UHM | Residuals | 2 |
| 2 | 27.17540526 | 13.58770263 | 10.6095163 | 3.36E-05 | UI | environment | 2 |
| 179 | 287.8623253 | 1.608169415 | 1.25568686 | 0.037027929 | UI | genotype | 2 |
| 352 | 450.809555 | 1.280708963 | NA | NA | UI | Residuals | 2 |
| 2 | 697.859434 | 348.929717 | 187.208225 | 4.20E-56 | STR | environment | 2 |
| 179 | 633.2949106 | 3.537960395 | 1.8981911 | 1.87E-07 | STR | genotype | 2 |
| 352 | 656.078335 | 1.863858906 | NA | NA | STR | Residuals | 2 |
| 2 | 271.5164777 | 135.7582388 | 486.345636 | 5.00E-102 | ELO | environment | 2 |
| 179 | 153.7903072 | 0.859163727 | 3.07790182 | 1.27E-19 | ELO | genotype | 2 |
| 352 | 98.25707582 | 0.27913942 | NA | NA | ELO | Residuals | 2 |
| 2 | 38.83983901 | 19.4199195 | 29.3470326 | 1.63E-12 | SFC | environment | 2 |
| 179 | 191.6238299 | 1.07052419 | 1.61775688 | 7.31E-05 | SFC | genotype | 2 |
| 352 | 232.9302508 | 0.661733667 | NA | NA | SFC | Residuals | 2 |
| 2 | 48.78194577 | 24.39097289 | 181.527027 | 5.51E-55 | MIC | environment | 3 |
| 181 | 43.56938726 | 0.240714847 | 1.79149272 | 0.00000178 | MIC | genotype | 3 |
| 354 | 47.56539324 | 0.134365518 | NA | NA | MIC | Residuals | 3 |
| 2 | 0.138593985 | 0.069296993 | 50.8927442 | 3.74E-20 | UHM | environment | 3 |
| 181 | 0.783213398 | 0.004327146 | 3.1779204 | 8.27E-21 | UHM | genotype | 3 |
| 354 | 0.482016362 | 0.001361628 | NA | NA | UHM | Residuals | 3 |
| 2 | 33.39153479 | 16.69576739 | 13.8634712 | 0.0000016 | UI | environment | 3 |
| 181 | 412.0521529 | 2.276531232 | 1.89033691 | 0.000000199 | UI | genotype | 3 |
| 354 | 426.3219186 | 1.204299205 | NA | NA | UI | Residuals | 3 |
| 2 | 723.0212515 | 361.5106258 | 157.818682 | 1E-49 | STR | environment | 3 |
| 181 | 879.6241842 | 4.859802123 | 2.12156299 | 9.59E-10 | STR | genotype | 3 |
| 354 | 810.8974178 | 2.290670672 | NA | NA | STR | Residuals | 3 |
| 2 | 168.1614374 | 84.08071872 | 410.113728 | 6.72E-93 | ELO | environment | 3 |
| 181 | 86.25431262 | 0.476543164 | 2.32439608 | 7.61E-12 | ELO | genotype | 3 |
| 354 | 72.57639144 | 0.205018055 | NA | NA | ELO | Residuals | 3 |
| 2 | 58.18892698 | 29.09446349 | 50.7220481 | 4.27E-20 | SFC. | environment | 3 |
| 181 | 178.7430044 | 0.987530411 | 1.72161845 | 0.00000798 | SFC. | genotype | 3 |
| 354 | 203.0564707 | 0.57360585 | NA | NA | SFC. | Residuals | 3 |
| 2 | 50.28298151 | 25.14149076 | 174.349864 | 4.7E-53 | MIC | environment | 4 |
| 176 | 80.75087894 | 0.458811812 | 3.18174359 | 2.4E-20 | MIC | genotype | 4 |
| 345 | 49.74947565 | 0.144201379 | NA | NA | MIC | Residuals | 4 |
| 2 | 0.173400791 | 0.086700396 | 53.0261041 | 8.31E-21 | UHM | environment | 4 |
| 176 | 1.330310107 | 0.00755858 | 4.62284 | 3.93E-34 | UHM | genotype | 4 |
| 345 | 0.564092668 | 0.001635051 | NA | NA | UHM | Residuals | 4 |
| 2 | 150.2609229 | 75.13046143 | 60.4588187 | 3.09E-23 | UI | environment | 4 |
| 176 | 587.3605017 | 3.337275578 | 2.68556502 | 2.79E-15 | UI | genotype | 4 |
| 345 | 428.7217273 | 1.242671673 | NA | NA | UI | Residuals | 4 |
| 2 | 905.096967 | 452.5484835 | 178.365157 | 6.45E-54 | STR | environment | 4 |
| 176 | 1304.539052 | 7.412153707 | 2.92138855 | 1.06E-17 | STR | genotype | 4 |
| 345 | 875.3347901 | 2.53720229 | NA | NA | STR | Residuals | 4 |
| 2 | 185.152594 | 92.57629699 | 303.120621 | 1.04E-76 | ELO | environment | 4 |
| 176 | 219.523626 | 1.247293329 | 4.0839863 | 3.56E-29 | ELO | genotype | 4 |
| 345 | 105.3667096 | 0.305410752 | NA | NA | ELO | Residuals | 4 |
| 2 | 113.9843157 | 56.99215786 | 89.6834574 | 4.33E-32 | SFC. | environment | 4 |
| 176 | 250.1383574 | 1.421240667 | 2.23647922 | 1.1E-10 | SFC. | genotype | 4 |
| 345 | 219.2410398 | 0.635481275 | NA | NA | SFC. | Residuals | 4 |
| 2 | 31.61888077 | 15.80944038 | 175.919683 | 4.02E-52 | MIC | environment | 5 |
| 162 | 36.14171731 | 0.22309702 | 2.48251401 | 2.81E-12 | MIC | genotype | 5 |
| 317 | 28.48795831 | 0.089867376 | NA | NA | MIC | Residuals | 5 |
| 2 | 0.128151751 | 0.064075875 | 45.349396 | 4.78E-18 | UHM | environment | 5 |
| 162 | 0.54532585 | 0.003366209 | 2.38241837 | 2.47E-11 | UHM | genotype | 5 |
| 317 | 0.44790128 | 0.001412938 | NA | NA | UHM | Residuals | 5 |
| 2 | 77.23102284 | 38.61551142 | 29.7328562 | 1.46E-12 | UI | environment | 5 |
| 162 | 327.8044866 | 2.023484485 | 1.55802606 | 0.000443306 | UI | genotype | 5 |
| 317 | 411.7033711 | 1.298748805 | NA | NA | UI | Residuals | 5 |
| 2 | 560.0060188 | 280.0030094 | 122.705604 | 3.42E-40 | STR | environment | 5 |
| 162 | 717.8080959 | 4.430914172 | 1.94175771 | 0.000000282 | STR | genotype | 5 |
| 317 | 723.3651185 | 2.281908891 | NA | NA | STR | Residuals | 5 |
| 2 | 137.7267379 | 68.86336896 | 320.644009 | 7.09E-77 | ELO | environment | 5 |
| 162 | 112.5120238 | 0.694518666 | 3.23384192 | 2.4E-19 | ELO | genotype | 5 |
| 317 | 68.08076046 | 0.214765806 | NA | NA | ELO | Residuals | 5 |
| 2 | 105.3337814 | 52.66689068 | 69.5184254 | 9.26E-26 | SFC. | environment | 5 |
| 162 | 173.7770476 | 1.072697825 | 1.41592303 | 0.004651241 | SFC. | genotype | 5 |
| 317 | 240.1579762 | 0.75759614 | NA | NA | SFC. | Residuals | 5 |
| 2 | 52.47019773 | 26.23509886 | 210.501462 | 1.37E-60 | MIC | environment | 6 |
| 179 | 32.08814224 | 0.179263364 | 1.43834794 | 0.002173548 | MIC | genotype | 6 |
| 347 | 43.24710723 | 0.124631433 | NA | NA | MIC | Residuals | 6 |
| 2 | 0.481134046 | 0.240567023 | 197.841272 | 4.6E-58 | UHM | environment | 6 |
| 179 | 0.490939384 | 0.002742678 | 2.25556654 | 5.51E-11 | UHM | genotype | 6 |
| 347 | 0.421938033 | 0.00121596 | NA | NA | UHM | Residuals | 6 |
| 2 | 73.95039011 | 36.97519506 | 28.4167366 | 3.72E-12 | UI | environment | 6 |
| 179 | 247.7019301 | 1.383809665 | 1.06350635 | 0.312767438 | UI | genotype | 6 |
| 347 | 451.5083087 | 1.301176682 | NA | NA | UI | Residuals | 6 |
| 2 | 814.1663111 | 407.0831555 | 194.34128 | 2.38E-57 | STR | environment | 6 |
| 179 | 651.878839 | 3.641781223 | 1.7385844 | 0.00000646 | STR | genotype | 6 |
| 347 | 726.8546084 | 2.094681868 | NA | NA | STR | Residuals | 6 |
| 2 | 238.5946054 | 119.2973027 | 547.359936 | 4.8E-108 | ELO | environment | 6 |
| 179 | 171.0999137 | 0.95586544 | 4.38570223 | 2.7E-32 | ELO | genotype | 6 |
| 347 | 75.62877968 | 0.217950374 | NA | NA | ELO | Residuals | 6 |
| 2 | 47.63021198 | 23.81510599 | 47.6929593 | 5.02E-19 | SFC | environment | 6 |
| 179 | 119.8917983 | 0.669786583 | 1.3413379 | 0.010721925 | SFC | genotype | 6 |
| 347 | 173.2717344 | 0.499342174 | NA | NA | SFC | Residuals | 6 |

Sum_Sq = Sum of squares, Df = degrees of freedom, Mean_Sq = Mean sum of squares, Pr(>F) = P-value

**Supplementary Table 3-** Table depicting the statistical significance of family factor in joint population of all individuals

| Trait | Name | df | SS | MS | F-value | pr>F |
| --- | --- | --- | --- | --- | --- | --- |
| MIC | family | 18 | 3.58014 | 0.1989 | 9.38 | 3.14E-24 |
| UHM | family | 18 | 0.27369 | 0.0152 | 29.49 | 2.27E-79 |
| UI | family | 18 | 23.59945 | 1.31108 | 12.87 | 1.19E-34 |
| STR | family | 18 | 217.6613 | 12.09229 | 22.65 | 6.04E-62 |
| ELO | family | 18 | 48.61054 | 2.70059 | 37.98 | 3.69E-99 |
| SFC. | family | 18 | 13.2666 | 0.73703 | 14.32 | 7.30E-39 |

df = degrees of freedom, SS = Sum of Squares, MS = Mean Sum of Squares

**Supplementary Table 4-** Table summarizing the QTL statistics identified via population-specific analyses

| **QTL_name** | **Trait** | **Chromosome** | **Position (bp)** | **p-value** | **PVE** | **Additive effect** | **Interval(pvalue<0.001)** | **Favorable allele source** | **Model** | **Population** |
| --- | --- | --- | --- | --- | --- | --- | --- | --- | --- | --- |
| qELO_pop2_A01 | ELO | A01 | 44209036 | 7.90E-06 | 0.354 | 0 - 0.183 | 16268468 - 108028857 | T257 | Nested | 2 |
| qELO_pop2_A01 | ELO | A01 | 90381325 | 1.67E-07 | 0.213 | 0.12 | 16268468 - 108028857 | T257 | Non-nested | 2 |
| qELO_pop2_D08 | ELO | D08 | 59475594 | 4.20E-07 | 0.198 | 0.098 | 56943119 - 59475594 | DES56 | Non-nested | 2 |
| qELO_pop2_D04 | ELO | D04 | 50252859 | 9.10E-06 | 0.15 | 0.086 | 47697119 - 54099982 | T257 | Non-nested | 2 |
| qELO_pop3_D04 | ELO | D04 | 53597804 | 2.00E-10 | 24.209 | 0.091 - 0.208 | 53597804 - 53860854 | T1046 | Nested | 3 |
| qELO_pop3_D04 | ELO | D04 | 53597804 | 1.58E-13 | 4.702 | 0.144 | 53597804 - 53860854 | T1046 | Non-nested | 3 |
| qELO_pop3_A01 | ELO | A01 | 43148650 | 3.82E-08 | 2.42 | 0.109 | 16087031 - 43148650 | T1046 | Non-nested | 3 |
| qELO_pop3_A01 | ELO | A01 | 43148650 | 1.36E-06 | 6.727 | 0.048 | 16087031 - 43148650 | T1046 | MLMM | 3 |
| qELO_pop3_D04 | ELO | D04 | 53597804 | 9.21E-15 | 30.993 | 0.075 | 53597804 - 53860854 | T1046 | MLMM | 3 |
| qELO_pop4_D11 | ELO | D11 | 68864680 | 1.06E-06 | 3.288 | 0.139 | 34694727 - 72522178 | DES56 | Non-nested | 4 |
| qELO_pop5_D01_1 | ELO | D01 | 1269117 | 2.44E-09 | 15.244 | 0.047 - 0.324 | 1269117 - 5714641 | DES56 | Nested | 5 |
| qELO_pop5_D01_1 | ELO | D01 | 1269117 | 4.34E-08 | 3.894 | 0.19 | 1269117 - 5714641 | DES56 | Non-nested | 5 |
| qELO_pop5_D01_2 | ELO | D01 | 53077828 | 2.66E-06 | 2.785 | 0.109 | 17257464 - 53077828 | T281 | Non-nested | 5 |
| qELO_pop6_A05 | ELO | A05 | 58498251 | 3.86E-09 | 0.702 | 0.093 - 0.27 | 48630550 - 58067056 | T063 | nested | 6 |
| qELO_pop6_D01 | ELO | D01 | 47945539 | 4.99E-07 | 0.525 | 0.032 - 0.210 | 42910334 - 48938296 | T063 | nested | 6 |
| qELO_pop6_A05 | ELO | A05 | 58498251 | 1.24E-09 | 0.344 | 0.316 | 48630550 - 58067056 | T063 | non-nested | 6 |
| qELO_pop6_D01 | ELO | D01 | 48025820 | 2.18E-07 | 0.243 | 0.816 | 42910334 - 48938296 | T063 | non-nested | 6 |
| qELO_pop6_D05 | ELO | D05 | 46813712 | 2.38E-06 | 0.198 | 1.31 | 32645789 - 58067056 | DES56 | non-nested | 6 |
| qMIC_pop1_A08 | MIC | A08 | 39086527 | 9.88E-09 | 19.462 | 0.066 - 0.091 | 1802098 - 108926582 | T326 | Nested | 1 |
| qMIC_pop1_A08 | MIC | A08 | 39086527 | 4.06E-10 | 2.067 | 0.074 | 1802098 - 108926582 | T326 | Non-nested | 1 |
| qMIC_pop2_D01 | MIC | D01 | 36997917 | 1.44E-05 | 0.123 | 0.035 | 29022511 - 60175439 | DES56 | Non-nested | 2 |
| qMIC_pop4_A11 | MIC | A11 | 122328186 | 1.37E-07 | 17.189 | 0.150- 0.175 | 119176754 - 122328186 | T1046 | Nested | 4 |
| qMIC_pop4_A11 | MIC | A11 | 122076153 | 3.32E-08 | 4.553 | 0.117 | 119176754 - 122328186 | T1046 | Non-nested | 4 |
| qMIC_pop4_D03 | MIC | D03 | 22007347 | 5.37E-06 | 2.995 | 0.098 | 12506104- 44099908 | T1046 | Non-nested | 4 |
| qMIC_pop4_A11 | MIC | A11 | 122076153 | 7.03E-08 | 31.248 | 0.059 | 119176754 - 122328186 | DES56 | MLMM | 4 |
| qMIC_pop5_D02 | MIC | D02 | 64131289 | 1.73E-05 | 1.501 | 0.069 | 64131289 - 66133825 | DES56 | Non-nested | 5 |
| qSFC._pop1_D06 | SFC | D06 | 6957599 | 2.74E-06 | 0.453 | 0.079 | * | T326 | Non-nested | 1 |
| qSFC._pop1_D12 | SFC | D12 | 43564507 | 1.34E-05 | 0.386 | 0.121 | 41994181 -48415766 | DES56 | Non-nested | 1 |
| qSFC_pop4_D04 | SFC | D04 | 21067784 | 9.44E-08 | 4.283 | 0.13- 0.229 | 21067784-26379211 | T1046 | Nested | 4 |
| qSFC_pop4_A13 | SFC | A13 | 48843925 | 4.86E-08 | 3.721 | 0.229 | 14693703 - 76269274 | DES56 | Non-nested | 4 |
| qSFC_pop4_A13 | SFC | A13 | 48843925 | 9.72E-06 | 33.497 | 0.112 | 14693703 - 76269274 | DES56 | MLMM | 4 |
| qSTR_pop1_A07 | STR | A07 | 44756684 | 5.93E-08 | 1.684 | 0 - 0.68 | 27358053 - 94227757 | T326 | Nested | 1 |
| qSTR_pop1_A07 | STR | A07 | 44756684 | 8.13E-09 | 1.742 | 0.68 | 27358053 - 94227757 | T326 | Non-nested | 1 |
| qSTR_pop1_A03 | STR | A03 | 59311294 | 3.46E-06 | 1.084 | 0.303 | 46891360 - 97617935 | T326 | Non-nested | 1 |
| qSTR_pop3_D02 | STR | D02 | 13810498 | 1.05E-07 | 9.319 | 0 - 0.533 | 13810498 - 41990115 | T1046 | Nested | 3 |
| qSTR_pop3_D02 | STR | D02 | 13810498 | 1.05E-07 | 0.89 | 0.533 | 13810498 - 41990115 | T1046 | Non-nested | 3 |
| qSTR_pop3_D02 | STR | D02 | 22388854 | 1.64E-06 | 20.516 | 0.209 | 13810498 - 41990115 | T1046 | MLMM | 3 |
| qSTR_pop4_D04 | STR | D04 | 13782765 | 9.50E-06 | 1.608 | 0.379 - 0.61 | 13782765- 35083328 | T1046 | Nested | 4 |
| qSTR_pop4_A13 | STR | A13 | 48843925 | 6.71E-06 | 1.488 | 0.445 | 14693703 - 76269274 | DES56 | Non-nested | 4 |
| qSTR_pop6_D01 | STR | D01 | 48285638 | 1.13E-06 | 0.017 | 0.183 | 43089736 - 61997127 | DES56 | non-nested | 6 |
| qUHM_pop1_A08 | UHM | A08 | 51171534 | 1.10E-07 | 0.786 | 0.009 | 25412189 - 94147879 | T326 | Non-nested | 1 |
| qUHM_pop1_A03 | UHM | A03 | 91727570 | 8.27E-06 | 0.538 | 0.007 | 52517711 - 103710581 | T326 | Non-nested | 1 |
| qUHM_pop1_D06 | UHM | D06 | 6957599 | 1.07E-05 | 36.033 | 0.004 | * | T326 | MLMM | 1 |
| qUHM_pop2_D01 | UHM | D01 | 63189430 | 3.69E-06 | 0.027 | 0.003 - 0.007 | 58277994 - 63189430 | T257 | Nested | 2 |
| qUHM_pop2_D01 | UHM | D01 | 63189430 | 3.05E-07 | 0.289 | 0.005 | 58277994 - 63189430 | T257 | Non-nested | 2 |
| qUHM_pop2_D01 | UHM | D01 | 63189430 | 6.32E-07 | 13.424 | 0.002 | 58277994 - 63189430 | T257 | MLMM | 2 |
| qUHM_pop4_A13 | UHM | A13 | 62146285 | 9.67E-09 | 1.643 | 0.017 | 12839269-75302908 | DES56 | Non-nested | 4 |
| qUHM_pop4_A12_1 | UHM | A12 | 107178868 | 3.33E-07 | 1.272 | 0.014 | 107178868 - 107575745 | T1046 | Non-nested | 4 |
| qUHM_pop4_D03 | UHM | D03 | 49322147 | 1.85E-06 | 1.099 | 0.011 | 47999318 - 50043473 | T1046 | Non-nested | 4 |
| qUHM_pop4_A12_2 | UHM | A12 | 7094350 | 1.16E-05 | 0.92 | 0.01 | 6923713 - 7094350 | T1046 | Non-nested | 4 |
| qUHM_pop5_A08 | UHM | A08 | 124663792 | 6.54E-06 | 0.602 | 0.01 | 123821276 -125467361 | DES56 | Non-nested | 5 |
| qUHM_pop5_A08 | UHM | A08 | 124663792 | 8.57E-06 | 17.155 | 0.005 | 123821276 -125467361 | DES56 | MLMM | 5 |

PVE = Phenotypic Variance Explained value, Nested = nested joint linkage association model, Non-nested = Non-nested joint linkage association model, MLMM = Multi-locus Mixed Linear Model.

**Supplementary Table 5-** Table summarizing the QTL statistics identified via joint analysis of all 6 populations

| **QTL_name** | **Trait** | **Chromosome** | **Position (bp)** | **p-vlaue** | **PVE** | **Additive effect** | **Interval (pvalue<0.0001)** | **Favorable allele source** |
| --- | --- | --- | --- | --- | --- | --- | --- | --- |
| qMIC_A11 | MIC | A11 | 120932319 | 6.1712E-16 | 5.351 | 0.112 | 119073078 - 121792143 | T1046 |
| qMIC_D03 | MIC | D03 | 8418950 | 4.4377E-08 | 2.407 | 0.088 | 7394342 - 41935761 | T1046 |
| qMIC_A08 | MIC | A08 | 51868732 | 3.0335E-07 | 2.104 | 0.069 | 7547024 - 78200437 | T326 |
| qUHM_D09 | UHM | D09 | 43030709 | 3.2258E-08 | 1.963 | 0.018 | 43030709 - 53225197 | DES56 |
| qUHM_D02_1 | UHM | D02 | 4526036 | 5.7944E-07 | 1.599 | 0.009 | 4006197 - 4569454 | T1046,T063 |
| qUHM_D02_2 | UHM | D02 | 31017663 | 6.5675E-07 | 1.583 | 0.007 | 31017663-41920332 | T1046,T063,T257 |
| qUI_A13 | UI | A13 | 13507504 | 4.1238E-10 | 3.204 | 0.261 | 12355368-46678267 | DES56 |
| qSTR_D02 | STR | D02 | 41641056 | 1.0197E-08 | 2.227 | 0.212 | 31017663-41920332 | T1046, T063,T256 |
| qSTR_A07 | STR | A07 | 41308176 | 4.9033E-08 | 2.017 | 0.841 | 40189644-44727245 | T326 |
| qSTR_D01 | STR | D01 | 48590025 | 5.724E-08 | 1.996 | 0.262 | 44290588 - 61369022 | DES56 |
| qSTR_D09 | STR | D09 | 45296673 | 1.3234E-07 | 1.884 | 0.572 | 43030709 - 45554618 | DES56 |
| qELO_D04 | ELO | D04 | 53747393 | 4.3608E-15 | 3.289 | 0.108 | 48460268 - 53747393 | T1046, T063, T257 |
| qELO_D01_1 | ELO | D01 | 1011588 | 2.9631E-10 | 2.098 | 0.159 | 449022 - 1525988 | DES56 |
| qELO_D01_2 | ELO | D01 | 44290588 | 2.7337E-10 | 2.106 | 0.091 | 16948473 - 48903721 | T1046, T281, T063,T326 |
| qELO_A01 | ELO | A01 | 87693325 | 4.6454E-10 | 2.051 | 0.125 | 25828900-108474950 | T1046, T257 |
| qELO_D11 | ELO | D11 | 68450513 | 6.8736E-10 | 2.009 | 0.151 | 64429869 - 72918108 | DES56 |
| qELO_A05 | ELO | A05 | 71399886 | 7.2747E-09 | 1.763 | 0.102 | 42379245 - 103018060 | T1046, T063, T257 |
| qSFC_A13 | SFC | A13 | 13507504 | 4.2263E-11 | 3.425 | 0.196 | 12355368 - 98939123 | DES56 |
| qSFC_A01 | SFC | A01 | 77901774 | 4.7381E-07 | 1.979 | 0.065 | 69367341 -97180300 | T1046, T063, T257,T281 |

PVE = Phenotypic Variance Explained Value

**Supplementary Table 6-** Broad sense heritabilities or plot level heritability values calculated for 6 fiber quality traits in all populations and the joint population

| **Population** | **MIC** | **UHM** | **UI** | **STR** | **ELO** | **SFC.** |
| --- | --- | --- | --- | --- | --- | --- |
| **1** | 0.289 | 0.35 | 0.066 | 0.344 | 0.239 | 0.11 |
| **2** | 0.213 | 0.425 | 0.232 | 0.276 | 0.311 | 0.198 |
| **3** | 0.423 | 0.55 | 0.363 | 0.394 | 0.511 | 0.293 |
| **4** | 0.335 | 0.319 | 0.159 | 0.24 | 0.431 | 0.119 |
| **5** | 0.133 | 0.294 | 0.028 | 0.201 | 0.538 | 0.107 |
| **6** | 0.175 | 0.254 | 0.077 | 0.233 | 0.411 | 0.171 |
| **Joint Population** | 0.301 | 0.473 | 0.18 | 0.337 | 0.521 | 0.186 |

**Supplementary Table 7 -** Summary table depicting the number of families showing superior mean transgressive predicted breeding values compared to the parents for all fiber quality traits

| **Trait** | **Population1** | **Population2** | **Population3** | **Population4** | **Population5** | **Population6** |
| --- | --- | --- | --- | --- | --- | --- |
| MIC | 0 | 2 | 0 | 0 | 4 | 0 |
| UHM | 2 | 0 | 1 | 1 | 0 | 0 |
| UI | 4 | 0 | 1 | 2 | 4 | 0 |
| STR | 4 | 0 | 0 | 1 | 1 | 0 |
| ELO | 0 | 1 | 0 | 1 | 0 | 0 |
| SFC | 4 | 0 | 1 | 1 | 4 | 0 |

1. ***Supplementary Figures with Captions***

**Supplementary Fig 1-** Mating design for each BC_1_F_2_ population in the present study

**
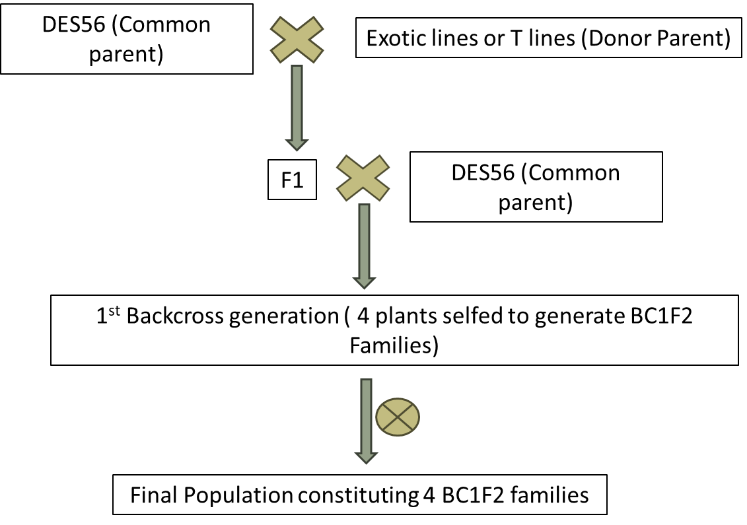
**

**Supplementary Fig 2A-** Venn Diagram depicting the number of QTLs detected via different methods in population-specific analysis. Nested = nested joint linkage association model, Non-nested = Non-nested joint linkage association model, MLMM = Multi-locus Mixed Linear Model.

**
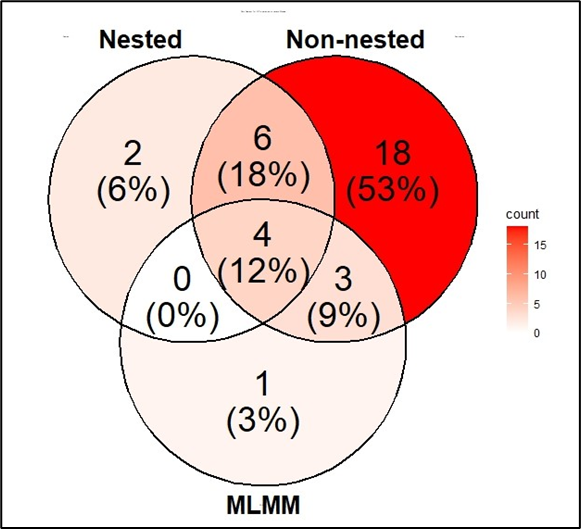
**

**Supplementary Figure 2B-** Distribution of PVE values for QTLs detected by three methods in population-specific analysis. PVE = Phenotypic Variance Explained value, Nested = nested joint linkage association model, Non-nested = Non-nested joint linkage association model, MLMM = Multi-locus Mixed Linear Model.

**
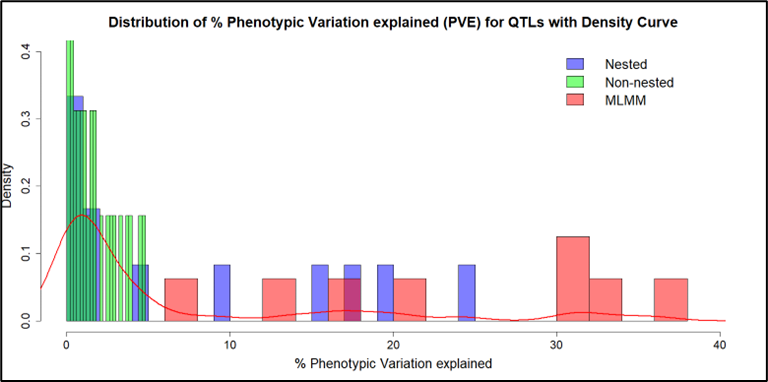
**
